# Genetic variants in adult bone mineral density and fracture risk genes are associated with the rate of bone mineral density acquisition in adolescence

**DOI:** 10.1093/hmg/ddv143

**Published:** 2015-05-04

**Authors:** Nicole M. Warrington, John P. Kemp, Kate Tilling, Jonathan H. Tobias, David M. Evans

**Affiliations:** 1 The University of Queensland Diamantina Institute, The University of Queensland, Translational Research Institute, Brisbane, Australia,; 2 MRC Integrative Epidemiology Unit,; 3 School of Social and Community Medicine and; 4 School of Clinical Sciences, University of Bristol, Bristol, UK

## Abstract

Previous studies have identified 63 single-nucleotide polymorphisms (SNPs) associated with bone mineral density (BMD) in adults. These SNPs are thought to reflect variants that influence bone maintenance and/or loss in adults. It is unclear whether they affect the rate of bone acquisition during adolescence. Bone measurements and genetic data were available on 6397 individuals from the Avon Longitudinal Study of Parents and Children at up to five follow-up clinics. Linear mixed effects models with smoothing splines were used for longitudinal modelling of BMD and its components bone mineral content (BMC) and bone area (BA), from 9 to 17 years. Genotype data from the 63 adult BMD associated SNPs were investigated individually and as a genetic risk score in the longitudinal model. Each additional BMD lowering allele of the genetic risk score was associated with lower BMD at age 13 [per allele effect size, 0.002 g/cm^2^ (SE = 0.0001, *P* = 1.24 × 10^−38^)] and decreased BMD acquisition from 9 to 17 years (*P* = 9.17 × 10^−7^). This association was driven by changes in BMC rather than BA. The genetic risk score explained ∼2% of the variation in BMD at 9 and 17 years, a third of that explained in adults (6%). Genetic variants that putatively affect bone maintenance and/or loss in adults appear to have a small influence on the rate of bone acquisition through adolescence.

## Introduction

Osteoporosis is characterized by loss of bone strength and increased risk of fracture. It is prevalent in 10% of women over the age of 50 years and 2% of men in the USA (1). Osteoporosis is defined by low bone mineral density (BMD), which is inversely related to the risk of osteoporotic fractures (2). Bone strength and fracture risk in the elderly are influenced by (i) peak bone mass attainment in adolescence and early adulthood, (ii) the subsequent maintenance of bone mass over the life course and (iii) the progressive loss of bone mass in later life (3).

BMD is a highly heritable quantitative trait, with heritability estimates ranging from 72 to 92% (4–7). Genome-wide association studies (GWAS) have successfully identified genetic loci associated with decreased BMD in adults, increased risk of osteoporotic fracture and increased risk of osteoporosis (8–17). The largest genome-wide meta-analysis to date on BMD of the femoral neck and lumbar spine included 32 961 individuals of European and East Asian descent, which confirmed 24 known loci and identified 32 novel loci for BMD (16). These identified loci are thought to influence bone maintenance and/or bone loss in adults, but it is unclear whether they might also have effects on peak bone mass.

Peak bone mass, which is thought to occur at the end of the skeletal maturation between late adolescence and early adulthood (18,19), is an important factor in determining future osteoporosis and long-term fracture risk (20). It is thought that individuals who have the highest peak bone mass are advantaged when bone density declines (20). Familial resemblance in BMD is present before puberty (21) and strengthens throughout adolescence and early adulthood (22,23). Genetic variants that influence bone maintenance and bone loss in adulthood may begin having an effect early in life. Kemp *et al*. (24) have shown that a subset of the loci associated with BMD in adults is also associated with BMD in childhood. There are also genetic variants in osteoblast-related genes, specifically *LRP5* and *ESR1*, and the Wnt signalling pathway (*WNT16*), that have been reported to be associated with BMD in both children (25–27) and adults (9,10,16,28). These studies provide additional evidence that there is a shared genetic influence on BMD in children and adults. In addition to variants that start having an effect in childhood, there may be a subset of variants that act on how rapidly bone acquisition accrues. These variants may not be detected in childhood but begin to show an association with BMD as it reaches its peak, an effect that may persist into adulthood.

The aim of this research is to examine the effect of 63 autosomal genetic variants, from 55 genetic loci, associated with adult BMD and fracture risk (16) on total body (excluding skull) BMD at 13 years of age and the rate of acquisition throughout adolescence.

## Results

**Figure 1.**
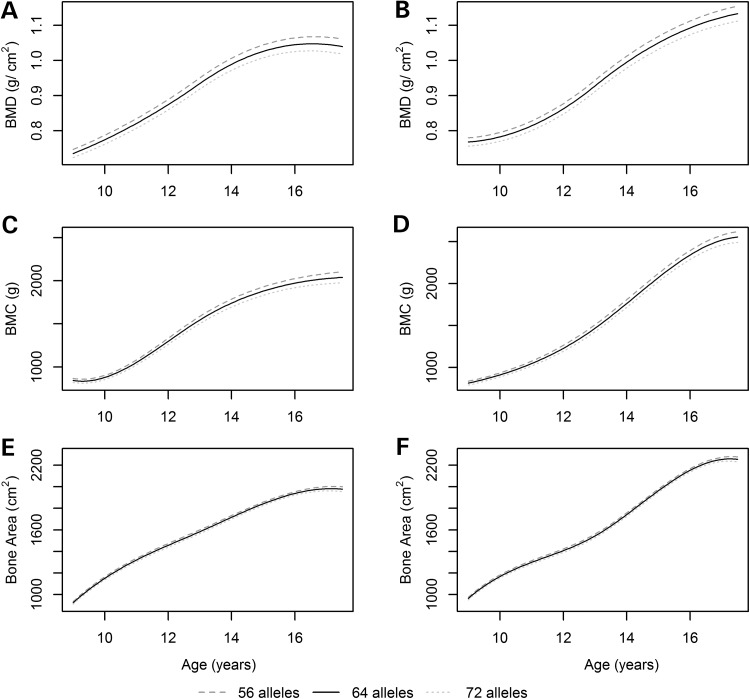
Population average curves of total body (excluding skull) BMD (**A**, **B**), BMC (**C**, **D**) and BA (**E**, **F**) for individuals with 56 (5th percentile), 64 (50th percentile) and 72 (95th percentile) BMD-lowering alleles in females (left panel) and males (right panel).

Bone measures and genotypes were available on 6397 individuals, consisting of 3233 females and 3164 males. A total of 20 424 BMD measures were available, with a median of three bone measurements per individual throughout the 8-year follow-up period. More bone measures were available from the earlier than the later follow-up years (Table 1). All three bone measures were higher in males than females at the 9-, 15- and 17-year follow-ups (Table 1). By the 17-year follow-up, female's total body (excluding skull) BMD trajectories were beginning to plateau, whereas the males were still increasing (Fig. 1). All 63 SNPs were common in this population (median BMD lowering allele frequency: 0.4, range: 0.07–0.72; Supplementary Material, Table S1) and they all imputed well (all *R*^2^ for imputation quality > 0.72). Individuals in this sample had an average of 64 BMD lowering alleles (range: 47–83 alleles).

**Table 1.** Basic characteristics of the ALSPAC sample used in the analysis

Follow-up	Number of individuals (% male)	All individuals [Mean (SD)]	Males [Mean (SD)]	Females [Mean (SD)]	*P*-value^a^
Age (years)					
Year 9	5289 (49)	9.91 (0.32)	9.91 (0.32)	9.91 (0.32)	0.74
Year 11	5113 (49)	11.78 (0.24)	11.78 (0.24)	11.78 (0.24)	0.36
Year 13	4083 (48)	13.87 (0.21)	13.86 (0.20)	13.88 (0.21)	0.07
Year 15	3047 (45)	15.47 (0.28)	15.46 (0.25)	15.48 (0.29)	0.05
Year 17	2892 (42)	17.81 (0.38)	17.81 (0.39)	17.80 (0.37)	0.71
BMD (g/cm^2^)					
Year 9	5289 (49)	0.78 (0.05)	0.78 (0.05)	0.77 (0.05)	<0.01
Year 11	5113 (49)	0.85 (0.07)	0.85 (0.06)	0.85 (0.07)	<0.01
Year 13	4083 (48)	1.00 (0.10)	0.99 (0.11)	1.01 (0.09)	<0.01
Year 15	3047 (45)	1.02 (0.09)	1.05 (0.10)	1.00 (0.07)	<0.01
Year 17	2892 (42)	1.08 (0.10)	1.14 (0.10)	1.04 (0.07)	<0.01
BA (cm^2^)					
Year 9	5289 (49)	1136.72 (161.60)	1146.51(152.75)	1127.32 (169.15)	<0.01
Year 11	5113 (49)	1415.94 (217.38)	1391.68 (200.71)	1439.05 (229.81)	<0.01
Year 13	4083 (48)	1664.10 (198.51)	1671.17 (213.59)	1657.56 (183.28)	0.03
Year 15	3047 (45)	1986.33 (262.59)	2084.24 (268.43)	1904.73 (227.50)	<0.01
Year 17	2892 (42)	2045.12 (259.64)	2196.55 (227.64)	1937.26 (225.48)	<0.01
BMC (g)					
Year 9	5289 (49)	889.63 (178.82)	902.29 (170.16)	877.48 (185.98)	<0.01
Year 11	5113 (49)	1214.65 (270.93)	1186.90 (242.53)	1241.09 (293.08)	<0.01
Year 13	4083 (48)	1683.59 (343.99)	1681.35 (376.39)	1685.66 (311.10)	0.69
Year 15	3047 (45)	2052.75 (419.31)	2214.18 (448.26)	1918.23 (339.34)	<0.01
Year 17	2892 (42)	2224.33 (454.21)	2515.05 (432.54)	2017.27 (342.02)	<0.01
Height (cm)					
Year 9	5289 (49)	139.60 (6.32)	139.87 (6.12)	139.35 (6.50)	<0.01
Year 11	5113 (49)	150.82 (7.23)	150.10 (7.09)	151.50 (7.29)	<0.01
Year 13	4082 (48)	162.89 (7.48)	164.22 (8.43)	161.66 (6.22)	<0.01
Year 15	3047 (45)	169.03 (8.34)	174.32 (7.56)	164.62 (6.09)	<0.01
Year 17	2892 (42)	170.68 (8.92)	178.20 (6.35)	165.33 (6.22)	<0.01
Weight (kg)					
Year 9	5289 (49)	34.67 (7.34)	34.38 (7.08)	34.94 (7.56)	<0.01
Year 11	5113 (49)	43.72 (9.78)	42.96 (9.49)	44.63 (9.96)	<0.01
Year 13	4079 (48)	52.83 (9.39)	52.89 (10.16)	52.78 (8.61)	0.72
Year 15	3047 (45)	60.53 (10.61)	63.20 (10.98)	58.31 (9.45)	<0.01
Year 17	2892 (42)	64.99 (11.33)	70.29 (10.99)	61.22 (9.98)	<0.01

^a^Comparing males to females.

From the linear mixed effects model, the genetic risk score was associated with BMD at age 13, where each ‘BMD lowering allele’ was associated with a lower BMD of 0.0019 g/cm^2^ (SE = 0.0001, *P* = 1.24 × 10^−38^; Table 2). In addition, the genetic risk score was associated with rate of change in BMD over time (Wald *P* = 9.17 × 10^−7^; Table 2), whereby each additional ‘BMD lowering allele’ was associated with approximately a 0.0002 g/cm^2^ per year (SE = 0.00005, *P* = 5.03 × 10^−5^; Table 2) slower BMD acquisition. The results from the models that were not adjusted for height and weight had increased standard errors, and in some cases a decreased estimate of the effect size, leading to a weaker association (Supplementary Material, Table S2). A similar pattern was seen for BMC, where the genetic risk score was associated with BMC at age 13 (β = −4.83 g, SE = 0.39, *P* = 1.47 × 10^−34^; Table 3) and rate of change in BMC over time (Wald *P* = 5.29 × 10^−16^; SNP by age interaction β = −0.72 g/year, SE = 0.13, *P* = 2.66 × 10^−8^; Table 3). In contrast, the genetic risk score was associated with BA at age 13 (β = −1.92 cm^2^, SE = 0.24, *P* = 2.66 × 10^−15^; Table 4), and showed a weaker association with BA over time (Wald *P* = 2.38 × 10^−4^; SNP by age interaction β = −0.04 cm^2^/year, SE = 0.10, *P* = 0.71; Table 4) than BMD or BMC. Figure 1 shows that the difference in BA between individuals with high and low genetic risk remains fairly stable across this age range, whereas the trajectories for BMD and BMC show a slight divergence in both males and females.

**Table 2.** Associations between the BMD genetic risk scores (GRS) and BMD from the linear mixed effects model, adjusting for height and weight

Effect	GRS of 63 SNPs		Children's GRS (8 SNPs)		Fracture GRS (16 SNPs)		RANK-RANKL-OPG pathway GRS (3 SNPs)		Mesenchymal stem cell differentiation pathway GRS (3 SNPs)		WNT signalling pathway GRS (8 SNPs)	
	Beta	*P*-value	Beta	*P*-value	Beta	*P*-value	Beta	*P*-value	Beta	*P*-value	Beta	*P*-value
Score	−0.0019 (0.0001)	1.24 × 10^−38^	−0.0033 (0.0004)	4.47 × 10^−15^	−0.0027 (0.0003)	2.47 × 10^−20^	−0.0013 (0.0006)	0.045	−0.0022 (0.0006)	5.35 × 10^−4^	−0.0031 (0.0004)	1.43 × 10^−13^
Age:Score	−0.0002 (5 × 10^−5^)	5.03 × 10^−5^	−0.0003 (0.0001)	0.013	−0.0002 (0.0001)	0.011	−0.0001 (0.0002)	0.585	−0.0003 (0.0002)	0.105	−0.0003 (0.0001)	0.044
Age^2^:Score	2 × 10^−5^ (4 × 10^−5^)	0.654	5 × 10^−5^ (0.0001)	0.659	0.0002 (0.0001)	0.063	−0.0002 (0.0002)	0.430	0.0003 (0.0002)	0.087	0.0002 (0.0001)	0.186
Age^3^:Score	1 × 10^−5^ (2 × 10^−5^)	0.484	3 × 10^−5^ (4 × 10^−5^)	0.449	5 × 10^−5^ (3 × 10^−5^)	0.107	−4 × 10^−5^ (7 × 10^−5^)	0.531	0.0001 (6 × 10^−5^)	0.121	6 × 10^−5^ (4 × 10^−5^)	0.198
Age^3^:Score (after age 13)	−1 × 10^−5^ (2 × 10^−5^)	0.638	−3 × 10^−5^ (6 × 10^−5^)	0.668	−7 × 10^−5^ (4 × 10^−5^)	0.097	7 × 10−5 (0.0001)	0.466	−0.0002 (0.0001)	0.095	−7 × 10^−5^ (6 × 10^−5^)	0.244
Global Wald test		9.14 × 10^−47^		2.59 × 10^−20^		1.25 × 10^−20^		0.154		0.023		1.34 × 10^−15^
Wald test for score by age interaction		9.17 × 10^−7^		0.032		0.005		0.706		0.096		0.075

‘:Score’ indicates the interaction between the genetic risk score and the polynomial function for age.

**Table 3.** Associations between the BMD genetic risk scores (GRS) and BMC from the linear mixed effects model, adjusting for height and weight

Effect	GRS of 63 SNPs		Children's GRS (8 SNPs)		Fracture GRS (16 SNPs)		RANK-RANKL-OPG pathway GRS (3 SNPs)		Mesenchymal stem cell differentiation pathway GRS (3 SNPs)		WNT signalling pathway GRS (8 SNPs)	
	Beta	*P*-value	Beta	*P*-value	Beta	*P*-value	Beta	*P*-value	Beta	*P*-value	Beta	*P*-value
Score	−4.829 (0.392)	1.47 × 10^−34^	−7.94 (1.103)	6.73 × 10^−13^	−6.129 (0.763)	1.15 × 10^−15^	−4.399 (1.680)	0.009	−5.235 (1.657)	0.002	−7.842 (1.104)	1.35 × 10^−12^
Age:Score	−0.719 (0.129)	2.66 × 10^−8^	−1.140 (0.362)	0.002	−0.852 (0.252)	7.14 × 10^−4^	−0.248 (0.550)	0.653	−0.688 (0.542)	0.204	−1.423 (0.364)	9.23 × 10^−5^
Age^2^:Score	0.147 (0.117)	0.212	0.396 (0.336)	0.239	0.190 (0.226)	0.402	0.888 (0.504)	0.078	1.501 (0.490)	0.002	0.493 (0.329)	0.134
Age^3^:Score	0.049 (0.039)	0.214	0.147 (0.112)	0.191	0.056 (0.076)	0.464	0.236 (0.169)	0.163	0.438 (0.164)	0.008	0.183 (0.111)	0.097
Age^3^:Score (after age 13)	−0.074 (0.059)	0.206	−0.205 (0.168)	0.222	−0.095 (0.113)	0.425	−0.418 (0.252)	0.097	−0.727 (0.245)	0.003	−0.238 (0.165)	0.149
Global Wald test		2.60 × 10^−37^		1.38 × 10^−13^		3.91 × 10^−15^		0.052		0.005		3.06 × 10^−12^
Wald test for score by age interaction		5.29 × 10^−16^		0.003		1.96 × 10^−6^		0.210		0.011		2.27 × 10^−4^

‘:Score’ indicates the interaction between the genetic risk score and the polynomial function for age.

**Table 4.** Associations between the BMD genetic risk scores (GRS) and BA from the linear mixed effects model, adjusting for height and weight

Effect	GRS of 63 SNPs		Children's GRS (8 SNPs)		Fracture GRS (16 SNPs)		RANK-RANKL-OPG pathway GRS (3 SNPs)		Mesenchymal stem cell differentiation pathway GRS (3 SNPs)		WNT signalling pathway GRS (8 SNPs)	
	Beta	*P*-value	Beta	*P*-value	Beta	*P*-value	Beta	*P*-value	Beta	*P*-value	Beta	*P*-value
Score	−1.922 (0.242)	2.66 × 10^−15^	−3.090 (0.684)	6.41 × 10^−6^	−2.179 (0.471)	3.85 × 10^−6^	−2.389 (1.036)	0.021	−2.056 (1.018)	0.043	−2.873 (0.680)	2.46 × 10^−5^
Age:Score	−0.038 (0.101)	0.706	0.064 (0.282)	0.819	−0.043 (0.196)	0.825	0.071 (0.429)	0.868	−0.008 (0.422)	0.985	−0.417 (0.284)	0.120
Age^2^:Score	0.155 (0.099)	0.117	0.310 (0.284)	0.275	0.034 (0.191)	0.857	0.895 (0.425)	0.035	0.844 (0.413)	0.041	0.238 (0.279)	0.393
Age^3^:Score	0.033 (0.034)	0.320	0.077 (0.096)	0.422	−0.0005 (0.065)	0.994	0.237 (0.144)	0.099	0.239 (0.140)	0.088	0.095 (0.095)	0.315
Age^3^:Score (after age 13)	−0.071 (0.050)	0.151	−0.154 (0.142)	0.279	−0.022 (0.096)	0.821	−0.421 (0.213)	0.048	−0.401 (0.207)	0.053	−0.135 (0.140)	0.335
Global Wald test		3.11 × 10^−19^		4.49 × 10^−7^		2.74 × 10^−8^		0.062		0.139		7.19 × 10^−5^
Wald test for score by age interaction		2.38 × 10^−4^		0.184		8.34 × 10^−3^		0.120		0.239		0.262

‘:Score’ indicates the interaction between the genetic risk score and the polynomial function for age.

Based on the linear mixed effects model adjusted for height and weight, the difference in BMD between individuals with 56 ‘BMD lowering alleles' (representative of the bottom 5% of individuals' genetic risk score in our sample) and individuals with 72 ‘BMD lowering alleles' (representative of the top 5% of individuals' genetic risk score in our sample) at age 9 is 0.024 g/cm^2^, whereas by age 17 it is 0.038 g/cm^2^. This is similar to the estimated effect size from the cross-sectional analysis of BMD presented in Supplementary Material, Table S5 (i.e. the coefficient at the two time points in Supplementary Material, Table S5 multiplied by 16, the difference in the number of alleles). The difference in BMC between individuals with 56 ‘BMD lowering alleles' and individuals with 72 ‘BMD lowering alleles' at age 9 is 43.92 g, in comparison to 111.76 g by age 17. Finally, the difference in BA between these individuals is 22.81 cm^2^ at age 9 and 32.26 cm^2^ at age 17. Again, these differences were similar to the effect sizes estimated in the cross-sectional analysis of BMC (Supplementary Material, Table S6) and BA (Supplementary Material, Table S7). This highlights that there is a change in BA over adolescence, but to a smaller magnitude than BMD and BMC. After adjusting for age, sex, height and weight in the cross-sectional analysis, the genetic risk score explained an additional 2.02% of the variation in BMD at the 9-year follow-up and 1.73% at the 17-year follow-up. The genetic risk score explained much less of the variation in BMC and BA, with 0.57% of BMC explained at the 9-year follow-up, 0.72% of BMC explained at the 17-year follow-up, 0.16% of BA explained at the 9-year follow-up and 0.22% of BA explained at the 17-year follow-up. The proportion of variance explained by each of the genetic risk scores at each of the cross-sectional time points are in Supplementary Material, Tables S5 (for BMD), S6 (for BMC) and S7 (for BA).

After adjusting for height and weight, the genetic risk scores that were made up of the SNPs that reached genome-wide significance in the GWAS of BMD in children or were associated with fracture risk were associated with BMD at age 13 (child genetic risk score: β = −0.0033 g/cm^2^, *P* = 4.47 × 10^−15^; fracture genetic risk score: β = −0.0027 g/cm^2^, *P* = 2.47 × 10^−20^; Table 2) and were also associated with rate of BMD acquisition (child genetic risk score: Wald *P* = 0.032; fracture genetic risk score: Wald *P* = 0.005; Table 2). As seen with the overall genetic risk score, there is stronger evidence for association between the child and fracture genetic risk scores and BMC than BA over this time period (Tables 3 and 4, respectively). At the majority of the follow-up times, both the child and fracture genetic risk scores explained slightly less than half of the variance in each of the bone measures than the genetic risk score containing all 63 SNPs (Supplementary Material, Tables S5–S7).

The genetic risk score containing the RANK-RANKL-OPG function SNPs was marginally associated with BMD at age 13 (β = -0.0013 g/cm^2^, *P* = 0.05) but was not associated with rate of change in BMD over adolescence (Wald *P* = 0.706; Table 2) after adjusting for height and weight. However, the genetic risk scores for the mesenchymal stem cell differentiation functional pathway and the WNT signalling function pathway were associated with BMD at age 13 (β = −0.0022 g/cm^2^, *P* = 5.35 × 10^−4^ and β = −0.0031 g/cm^2^, *P* = 1.43 × 10^−13^ respectively; Table 2) and showed a weak association with rate of change in BMD over this age range (Wald *P* = 0.096 and Wald *P* = 0.075, respectively; Table 2). The WNT signally pathway genetic risk score explained a similar proportion of the variance in the bone measures as the child and fracture genetic risk scores; the genetic risk scores for the other two pathways explained a much smaller proportion of the variance in the bone measures at each follow-up (Supplementary Material, Tables S5–S7).

Results from the association analysis between each individual SNP and BMD are presented in Supplementary Material, Table S8. After adjusting for skeletal size, 11 individual SNPs showed significant association (Bonferonni *P*-value adjusting for 63 SNP: *P* < 7.94 × 10^−4^) with BMD at age 13, including rs9921222 (*AXIN1*), rs7851693 (*FUBP3*), rs4233949 (*SPTBN1*), rs13204965 (*RSPO3*), rs7812088 (*ABCF2*), rs3801387 (*WNT16*), rs13245690 (*C7orf58*), rs6426749 (*ZBTB40*), rs7521902 (*WNT4*), rs12407028 (*WLS*) and rs1286083 (*RSP6KA5*). Six of these SNPs (rs9921222, rs7851693, rs13204965, rs3801387, rs6426749 and rs7521902), in addition to rs4869742 (*C6orf97*), rs1026364 (*KIAA2018*) and rs7751941 (*ESR1*), were also significant for the BMD global Wald test. Two of these SNPs, rs4869742 (*C6orf97*) and rs6426749 (*ZBTB40*), also showed significant association for the Wald test of the SNP by age interaction. Association analysis between each of the 63 individual SNPs and BMC (Supplementary Material, Table S9) also showed a significant association with rs3801387 (*WNT16*), rs7851693 (*FUBP3*), rs13245690 (*C7orf58*) and rs9921222 (*AXIN1*), in addition to rs3736228 (*LRP5*). Three of these SNPs showed significant association with the global Wald test (rs9921222, rs3801387 and rs13245690) and one with the Wald test of the SNP by age interaction (rs9921222). Fewer SNPs were associated with BA, with one SNP associated at age 13 (rs3801387; *WNT16*), four with the global Wald test (rs13245690; *C7orf58*, rs4792909; *SOST*, rs3801387; *WNT16* and rs3736228; *LRP5*) and one with the Wald test for change over time (rs4792909; *SOST;* Supplementary Material, Table S10).

## Discussion

We investigated the association between the rate of change in BMD during adolescence and genetic variants thought to affect bone maintenance and/or loss in adults. We have shown that each additional BMD lowering allele in a genetic risk score of the 63 adult BMD SNPs was associated with decreased BMD at 13 years of age and also with a small decrease in the rate of change between 9 and 17 years, after adjusting for skeletal size (i.e. adjusting for height and weight). These associations with BMD seem to be driven by an association with in BMC rather than with BA. This could be expected as BMD is a measure of BMC adjusted for BA, so changes in BMC are more likely to be detected in BMD. The original paper reported that these 63 SNPs explain ∼6% of the variation in adult BMD; we have shown that from age 9 to 17, the same set of SNPs explains ∼2% of BMD variation.

The subset of SNPs that have been shown to be associated with BMD in children (24) were associated with BMD at 13 years of age and with rate of change over this period, only after adjustment for skeletal size was made. Each additional risk allele of this childhood genetic risk score was associated with decreased BMC and BA at age 13. It was also associated with rate of change in BMC between 9 and 17 years and showed weaker evidence of association with rate of change in BA over this time period. Hence, through adjusting BMD for skeletal size, we likely removed some error variance to detect the association with rate of change in BMD, which is primarily driven by change in BMC. Similarly, the genetic risk score comprising variants known to influence fracture risk in adults (16) was also associated with all three bone measures at 13 years of age and rate of change of BMD, only after adjustment for skeletal size was made. Both of these genetic risk scores appear to have an influence on BMD from early in life which persists through the life course. We also investigated genetic risk scores comprising variants that belonged to certain genetic pathways. These scores were associated with all three bone measures at 13 years of age, suggesting these pathways influence not only determinants of BMD such as cortical thickness and density, and trabecular bone volume, which is well recognized, but also overall bone size. The association between the RANK-RANKL-OPG pathway genetic risk score and bone size is consistent with our recent observations in ALSPAC that these markers are related to periosteal expansion as measured by pQCT (29). Presumably, mesenchymal stem cell differentiation and WNT signally contribute to bone size by influencing the supply of osteoblasts during periosteal bone formation. In contrast, these scores did not show strong evidence for association with change in trajectory over adolescence; however, this could be due to the smaller number of SNPs that were included in the scores (i.e. only three SNPs in the RANK-RANKL-OPG pathway score and mesenchymal stem cell differentiation functional pathway score) or it could be that the function of these SNPs does not influence change in BMD over this age. Further investigation, with larger sample sizes, is required to determine the cause of this lack of association.

Although we have lower power to detect associations with individual SNPs, we detected an association between 14 individual SNPs and BMD during adolescence after adjusting for skeletal size, indicating that there is overlap between genetic variants related to BMD in adults and BMD in adolescence. Of the 16 variants that were associated with increased fracture risk in adults, six were associated with BMD over adolescence and a further two were associated with BA. This indicated that half of those SNPs that increase fracture risk have detectable effects on bone acquisition in adolescence. Additionally, four of these, rs13204965, rs381387, rs13245690 and rs7521902, have previously been shown to be associated with BMD in childhood (24), indicating that their effect begins early in life and persists throughout the life course. The *AXIN1*, *WNT16* and *ZBTB40* genes, along with *RSPO3* and *WNT4*, belong to the WNT signalling pathway, a pathway which is involved in development and cell growth, and is well known to be involved in regulating BMD (30). The *WLS* gene plays a critical role in Wnt regulation and is required for intramembranous and endochondral ossification (31).

It has been shown that from 9 to 17 years BMD more than doubles in both males and females (32,33). The present results indicate that some of the rate of change is under genetic control. Peak bone mass is an important clinical phenotype as it has been shown to associate with fracture risk in later life (20). In terms of the mechanisms by which peak bone is achieved, those individuals with a higher BMD in early adolescence will show a greater subsequent gain, assuming they remain on the same BMD percentile. However, the trajectory of BMD gain is influenced by factors besides the starting value in early adolescence, including age of puberty, skeletal age and age of peak height velocity. For example, an individual with a relatively young age of peak height velocity is expected to have an early rapid gain in BMD which reaches a plateau within a short period of time, in contrast to an individual with a relatively late age of peak height velocity who will show a slower BMD gain that is maintained for longer. Therefore, although a high correlation exists between the BMD at age 13 years and trajectory from 9 to 17 years, these two parameters also represent distinct characteristics, justifying separate analysis of their associations with genetic risk scores. Although we could have examined genetic influences on BMD trajectory independently of the intercept by conditioning change in BMD on BMD at age 13 years (34–36), this would require a different class of statistical models to be fit to the data, which is beyond the scope of the current study. Subsequent bone measurements obtained through further follow-up of ALSPAC individuals will enable the estimation of peak bone mass that can be tested for association with these adult BMD SNPs and also enable more robust estimation of rate of change effects conditional on baseline BMD.

It is only by studying BMD throughout the life course that one can completely understand the factors influencing bone strength and consequently future risk of osteoporosis. The continual follow-up of the ALSPAC cohort, and cohorts similar to this, will facilitate the detection of genetic variants associated with the rate of BMD acquisition, which will aid in our understanding of the biological pathways underpinning attainment of peak bone mass.

## Materials and Methods

### Participants

The Avon Longitudinal Study of Parents and Children (ALSPAC) is a prospective cohort study. The full study methodology is published elsewhere (37) and the study website contains details of all the data that is available through a fully searchable data dictionary (www.bristol.ac.uk/alspac/researchers/data-access/data-dictionary/). Pregnant women resident in one of three Bristol-based health districts with an expected delivery date between 1 April 1991 and 31 December 1992 were invited to participate. Follow-up included parent and child completed questionnaires, links to routine health care data and clinic attendance. Individuals were included in this study based on the following criteria: live singleton birth, unrelated to anyone else in the sample (<10% identical by descent as calculated from genome-wide data), genotype data available and DXA data from at least one follow-up available. Ethical approval for the study was obtained from the ALSPAC Law and Ethics Committee and the Local Research Ethics Committees.

### Phenotypic variables

Total body DXA scans were performed on participants at five follow-ups (9-, 11-, 13-, 15- and 17-years) using a Lunar Prodigy scanner (Lunar Radiation Corp, Madison, WI, USA) with paediatric scanning software (GE Healthcare Biosciences Corp., Piscataway, NJ, USA). Scans were excluded if any anomalies were present (e.g. missing parts of limbs, movement artefacts). Further details of the measures, including reproducibility, are described elsewhere (38). DXA measures investigated included total body (excluding skull) BMD (g/cm^2^), and its components, bone mineral content (BMC, g) and bone area (BA, cm^2^). Total body (excluding skull) measures are preferred for paediatric evaluations of bone health as the variation during skeletal development is lower than the commonly used femoral neck or lumbar spine measurements in adults, therefore increasing reproducibility (39).

Height was measured to the nearest 0.1 cm using a Harpenden Stadiometer (Holtain Ltd. Crymych, UK). Weight was measured to the nearest 0.1 kg using the Tanita Body Fat Analyser (Tanita UK Ltd., Uxbridge).

### Genotyping and genetic risk score

Imputed genotypic data has been previously described (24). Briefly, ALSPAC individuals were genotyped using the Illumina HumanHap550 quad genome-wide single-nucleotide polymorphism (SNP) genotyping platform by the Wellcome Trust Sanger Institute, Cambridge, UK and the Laboratory Corporation of America, Burlington, NC, US. Genotype data was cleaned using standard thresholds (minor allele frequency (MAF) >1%, call rate >95% and *P*-value from an exact test of Hardy–Weinberg equilibrium >5 × 10^−7^). Individual samples were excluded on the basis of incorrect gender assignment, minimal or excessive heterozygosity, high levels of missingness or cryptic relatedness. Imputation of un-typed or missing genotypes was performed using MACH v1.0.16 using the samples from the CEU population in HapMap Phase2 (Build 36, release 22) as a reference panel. No obvious population stratification has been observed and genome-wide analyses with other phenotypes indicate a low lambda in the ALSPAC cohort, so no adjustment was made in the subsequent analysis.

Estrada *et al*. (16) reported that 63 autosomal SNPs were associated with adult BMD at genome-wide levels of significance. These 63 SNPs comprised 55 autosomal SNPs and eight secondary SNPs at these loci. Sixteen of these SNPs (14 autosomal SNPs and 2 secondary SNPs) were also associated with fracture risk. We extracted these 63 SNPs from the imputed data. All SNPs imputed well (all *R*^2^ for imputation quality > 0.72, mean = 0.978; Supplementary Material, Table S1), therefore, dosages from the imputed data were used in subsequent analyses (i.e. the estimated number of BMD-lowering alleles). An unweighted ‘genetic risk score’ was created by summing the dosages for the BMD-lowering alleles across all 63 SNPs. The distribution of the genetic risk score is presented in Supplementary Material, Figure S1 with the mean BMD at each score. A sensitivity analysis was conducted whereby the alleles were weighted by the effect sizes from the stage 2 meta-analysis for femoral neck in Estrada *et al*. (16); these were the same weights used in their allele risk modelling for osteoporosis and fracture. The weighted score gave the same conclusions as the unweighted score; therefore only the unweighted score is presented.

Five subsets of SNPs were also summed to create additional genetic risk scores:

1. Those SNPs that met genome-wide significance for BMD at any location (total body excluding skull, skull, lower limbs or upper limbs) in childhood (24). These included rs3801387 (*WNT16*), rs1346004 (*GALNT3*), rs13245690 (*CPED1*), rs7521902 (*WNT4*), rs13204965 (*RSPO3*), rs2062377 (*TNFRSF11B*), rs10835187 (*LIN7C*) and rs884205 (*TNFRSF11A*).
2. Those SNPs that were associated with increased risk of low-trauma fracture (16). These included rs4233949 (*SPTBN1*), rs6532023 (*MEPE/SPP1*), rs4727338 (*SLC25A13*), rs1373004 (*MBL2/DKK1*), rs3736228 (*LRP5*), rs4796995 (*FAM210A*), rs6426749 (*ZBTB40*), rs7521902 (*WNT4*), rs430727 (*CTNNB1*), rs6959212 (*STARD3NL*), rs3801387 (*WNT16*), rs7851693 (*FUBP3*), rs163879 (*DCDC5*), rs1286083 (*RPS6KA5*), rs4792909 (*SOST*) and rs227584 (*C17orf53*).
3. Those SNPs that were part of the RANK-RANKL-OPG pathway (16). These included rs884205 (*RANK*), rs9533090 (*RANKL*) and rs2062377 (*OPG*).
4. Those SNPs that were part of the mesenchymal stem cell differentiation functional pathway (16). These included rs2016266 (*SP7*), rs7217932 (*SOX9*) and rs11755164 (*SUPT3H*/*RUNX2*).
5. Those SNPs that were part of the Wnt signalling pathway (16). These included rs6959212 (*SRARD3NL*), rs9921222 (*AXIN1*), rs430727 (*CTNNB1*), rs2887571 (*ERC1*/*WNT5B*), rs1864325 (*MAPT*), rs3801387 (*WNT16*), rs6426749 (*ZBTB40*) and rs3736228 (*LRP5*).

### Statistical analyses

Linear mixed effects models, including a polynomial function for age, were used to investigate the association between the adult BMD SNPs and total body (excluding skull) BMD, BMC and BA acquisition during adolescence. We followed the guidelines in Cheng *et al*. (40) to select the most appropriate linear mixed model, using AIC and visual inspection of diagnostic plots (such as fitted versus observed values, fitted versus residual values and distribution of the random effects and error terms) to compare models. The final model included a knot point at 13 years in the fixed effects for all three phenotypes in addition to a cubic function of age for BMD, BMC and BA, which produced a smooth curve. The random effects included an intercept and a linear slope. Sex was included as a fixed effect in addition to an age by sex interaction, to allow males and females to have different intercepts and trajectories. Height, weight and their interactions with age were included to account for skeletal size. Models without adjustment for skeletal size were also conducted for completeness, and the results are presented in the online Supplementary Material; the models for BMC and BA without adjustment for skeletal size are the same; however, the model for BMD only includes a quadratic function for age. Additional details on the models used, including the mathematical equations, are included in the online Supplementary Material. We also investigated whether adjusting for puberty provided a better fit of the model to the data (results not shown); however, due to the large amount of missing data and potential inaccuracies in the exact timing of puberty, we determined that including a knot point at 13 years provided the best fit. All individuals with at least one measure of BMD, BA or BMC were included in these models. The predicted curves for BMD, BA and BMC are plotted in Supplementary Material, Figures S2–S4.

The genetic risk scores were tested for association with the rate of change in total body (excluding skull) BMD, BA and BMC, by including a main effect and an interaction between the age function and the score in the fixed effects part of the model. This estimates whether the score shifts the whole trajectory up or down (i.e. the main effect from the model, described here as the effect at the mean centred age of 13 years) or changes the shape of the trajectory (i.e. the effects estimated by the age function by score interaction terms in the model). In addition to the fixed effects estimates, a Wald test was conducted to test the association of the genetic risk scores with overall BMD, BA and BMC level and growth over this time period (referred to as the ‘global Wald test’). The null hypothesis was that the fixed effects estimated for the genetic risk score are simultaneously equal to zero. A statistically significant *P*-value from this global Wald test will indicate that each additional adult BMD lowering allele shifts the whole trajectory up/down from the population average and/or changes the shape of the trajectory. A second Wald test was also conducted to test the association of the genetic risk scores with growth of the three bone measures (i.e. excluding the main effect of the genetic risk score). The null hypothesis for this second test was that the fixed effects estimated for the genetic risk score by age interactions are simultaneously equal to zero (referred to as the ‘Wald test’). A statistically significant *P*-value from this Wald test will indicate that each additional adult BMD lowering allele changes the shape of the trajectory over adolescence, but will not provide any information on whether it shifts the trajectory up/down as it does not include the main effect of the genetic risk score. These two Wald tests were estimated using the General Linear Hypothesis approach (41,42). Additional details regarding the specific coefficients being tested are included in the online Supplementary Material.

Univariate linear models at each of the follow-up years were also conducted, adjusting for age, sex, height, weight and each of the genetic risk scores, and the results were used to aid in the interpretation of the longitudinal results.

All analyses were conducted in *R* (version 3.0.2 [2013-09-25]) (43) using the nlme and spida packages. The R code used for the cross-sectional and longitudinal models is included in the online Supplementary Material.

## Supplementary Material

Supplementary Material is available at *HMG* online.

## Authors' Roles

Study design: J.T., D.M.E. Data collection: J.T. Data analysis: N.M.W. Data interpretation: N.M.W., J.P.K., K.T., J.H.T. and D.M.E. Drafting manuscript: N.M.W., J.T., D.M.E. Revising manuscript content and approving final version: N.M.W., J.P.K., K.T., J.H.T. and D.M.E. N.M.W. takes responsibility for the integrity of the data analysis.

## Funding

This work was supported by the Medical Research Council MC_UU_12013/4 to D.M.E. D.M.E. is funded by an Australian Research Council Future Fellowship (FT130101709). The UK Medical Research Council and the Wellcome Trust (Grant ref: 102215/2/13/2) and the University of Bristol provide core support for ALSPAC. GWAS data was generated by Sample Logistics and Genotyping Facilities at the Wellcome Trust Sanger Institute and LabCorp (Laboratory Corporation of America) using support from 23andMe. Funding to pay the Open Access publication charges for this article was provided by the Wellcome Trust.

## Supplementary Material

Supplementary Data
