# Supplementary material for: Genetic variants in adult bone mineral density and fracture risk genes are associated with the rate of bone mineral density acquisition in adolescence
Source: Hum Mol Genet. 2015 May 4;24(14):4158–66. doi: 10.1093/hmg/ddv143 (PMC4476449; doi:10.1093/hmg/ddv143)
Supplement: Supplementary Data [file supp_24_14_4158__index.html]

Genetic variants in adult bone mineral density and fracture risk genes are associated with the rate of bone mineral density acquisition in adolescence — Genetic variants in adult bone mineral density and fracture risk genes are associated with the rate of bone mineral density acquisition in adolescence — Supplementary Data 

# Genetic variants in adult bone mineral density and fracture risk genes are associated with the rate of bone mineral density acquisition in adolescence

## Supplementary Data

Supplementary Data

**Files in this Data Supplement:**

- Supplementary Data - Docx file
