# Supplementary material for: Genetic variants in adult bone mineral density and fracture risk genes are associated with the rate of bone mineral density acquisition in adolescence
Source: Hum Mol Genet. 2015 May 4;24(14):4158–66. doi: 10.1093/hmg/ddv143 (PMC4476449; doi:10.1093/hmg/ddv143)
Supplement: Supplementary Data [file supp_ddv143_ddv143supp.docx]

**Supplementary Material**

**Supplementary Methods:**

The following linear mixed effects model for the j^th^ individual and at the t^th^ time-point was used to investigate the association between adult BMD SNPs and total body (excluding skull) BMD, BMC and BA:

|  | ${Bone}_{jt}=\beta_{0}+\beta_{1}{Age}_{jt}+\beta_{2}{Age}_{jt}^{2}+\beta_{3}{Age}_{jt}^{3}+\beta_{4}{({Age}_{jt}-\kappa_{1})}_{+}^{3}+\beta_{5}Sex+\beta_{6}{Age}_{jt}Sex+\beta_{7}{Age}_{jt}^{2}Sex+\beta_{8}{Age}_{jt}^{3}Sex+\beta_{9}{({Age}_{jt}-\kappa_{1})}_{+}^{3}Sex+\beta_{10}{SNP+\beta_{11}Age}_{jt}SNP+\beta_{12}{Age}_{jt}^{2}SNP+\beta_{13}{Age}_{jt}^{3}SNP+\beta_{14}{({Age}_{jt}-\kappa_{1})}_{+}^{3}SNP+\sum_{l} \mathrm{Covariate}_{l}+b_{0j}+b_{1j}{Age}_{jt}+\varepsilon_{\mathrm{jt}}$ |  |
| --- | --- | --- |

Where:

- Bone is the bone measure being analysed; either BMD, BMC or BA.
- Age was centred at 13 years.
- κ_k_ is the k^th^ knot and (Age - κ_k_)_+_ = 0 if Age ≤ κ_k_ and (Age - κ_k_) if Age > κ_k_, which is known as the truncated power basis that ensures smooth continuity between the time windows (4).
- sex is coded as 1 for male and 2 for female.
- Covariate_l_ is the l^th^ covariate which includes height, weight and their interaction with age.
- $b_{0j}$ and $b_{1j}$ are the random effects.

A series of models were fit with knot points placed at 6-month intervals around the visually estimated turning points; the model with the lowest Akaike Information Criterion was selected. These models allow for individual variation in growth trajectories through random effects that allow person-specific intercepts and slopes. They also allow for the adjustment of confounders, appropriately account for the correlation structure between repeated measures within an individual and allow for incomplete data assuming data are missing at random.

As described in the methods section of the manuscript, we used three tests to describe the results of the analysis. The coefficients in model (1) that are tested in each of the tests are as follows:

1. Global test (Wald test): The null hypothesis was that the four coefficients estimated for the SNP, $\beta_{10}$- $\beta_{14}$, are simultaneously equal to zero in equation (1).
2. SNP by age interaction (Wald test): Given the spline function has multiple parameters (i.e., it is a non-linear function of time), it is necessary to use a test that summarizes the effect of each SNP on BMD acquisition simultaneously. The null hypothesis was that the three coefficients estimated for the SNP by spline interaction, $\beta_{11}$- $\beta_{14}$ , are simultaneously equal to zero in equation (1).
3. SNP main effect: The null hypothesis was that $\beta_{10}$ was equal to zero in equation (1). It is interpreted as the effect of the SNP on BMD, BMC or BA at age 13, as that is the age at which our model was centered.

**R code for conducting longitudinal analysis:**

library(nlme)

library(spida)

# Create knot point at 13 years of age

data$age_g13 <- ifelse(data$age>=13, 1, 0)

data$age_g13 <- replace(data$age_g13, is.na(data$age>=13), NA)

table(data$age_g13, exclude=NULL)

# Cross-sectional models at each follow-up – example using BMD

summary(lm(tblh_bmd ~ age + kz021 + height + weight + score,

data[which(data$time==9),]))

summary(lm(tblh_bmd ~ age + kz021 + height + weight + score,

data[which(data$time==11),]))

summary(lm(tblh_bmd ~ age + kz021 + height + weight + score,

data[which(data$time==13),]))

summary(lm(tblh_bmd ~ age + kz021 + height + weight + score,

data[which(data$time==15),]))

summary(lm(tblh_bmd ~ age + kz021 + height + weight + score,

data[which(data$time==17),]))

# Calculating R-squared of the score

(summary(lm(tblh_bmd ~ age + kz021 + height + weight + score,

data[which(data$time==9),]))$adj.r.squared –

summary(lm(tblh_bmd ~ age + kz021 + height + weight, data[which(data$time==9),]))$adj.r.squared)

(summary(lm(tblh_bmd ~ age + kz021 + height + weight + score,

data[which(data$time==11),]))$adj.r.squared –

summary(lm(tblh_bmd ~ age + kz021 + height + weight,

data[which(data$time==11),]))$adj.r.squared)

(summary(lm(tblh_bmd ~ age + kz021 + height + weight + score,

data[which(data$time==13),]))$adj.r.squared –

summary(lm(tblh_bmd ~ age + kz021 + height + weight, data[which(data$time==13),]))$adj.r.squared)

(summary(lm(tblh_bmd ~ age + kz021 + height + weight + score,

data[which(data$time==15),]))$adj.r.squared –

summary(lm(tblh_bmd ~ age + kz021 + height + weight, data[which(data$time==15),]))$adj.r.squared)

(summary(lm(tblh_bmd ~ age + kz021 + height + weight + score,

data[which(data$time==17),]))$adj.r.squared –

summary(lm(tblh_bmd ~ age + kz021 + height + weight, data[which(data$time==17),]))$adj.r.squared)

# Longitudinal model without adjusting for skeletal size

bmd.tblh.score <- lme(tblh_bmd ~ (I(age-13) + I((age-13)^2) +

age_g13:I((age-13)^2))*(sex + score), data,

random=~ I(age-13) |ID, method="ML", na.action=na.omit)

summary(bmd.tblh.score)

wald(bmd.tblh.score, "score") # Global Wald Test

wald(bmd.tblh.score, "score")$score$anova$"p-value"

wald(bmd.tblh.score, ":score") # Wald Test for age by score interaction

wald(bmd.tblh.score, ":score")[[1]]$anova$"p-value"

bmc.tblh.score <- lme(tblh_bmc ~ (I(age-13) + I((age-13)^2) +

I((age-13)^3) + age_g13:I((age-13)^3))*(kz021 + score), data, random=~ I(age-13)|ID, method="ML", na.action=na.omit)

summary(bmc.tblh.score)

wald(bmc.tblh.score, "score") # Global Wald Test

wald(bmc.tblh.score, "score")$score$anova$"p-value"

wald(bmc.tblh.score, ":score") # Wald Test for age by score interaction

wald(bmc.tblh.score, ":score")[[1]]$anova$"p-value"

ba.tblh.score <- lme(tblh_ba ~ (I(age-13) + I((age-13)^2) + I((age-13)^3)

+ age_g13:I((age-13)^3))*(kz021 + score), data,

random=~ I(age-13)|ID, method="ML", na.action=na.omit)

summary(ba.tblh.score)

wald(ba.tblh.score, "score") # Global Wald Test

wald(ba.tblh.score, "score")$score$anova$"p-value"

wald(ba.tblh.score, ":score") # Wald Test for age by score interaction

wald(ba.tblh.score, ":score")[[1]]$anova$"p-value"

# Longitudinal model adjusting for height and weight

bmd.tblh.hw.score <- lme(tblh_bmd ~ (I(age-13) + I((age-13)^2) +

I((age-13)^3) + age_g13:I((age-13)^3))*(kz021 + height + weight + score), data, random=~ I(age-13) |ID, method="ML", na.action=na.omit)

summary(bmd.tblh.hw.score)

wald(bmd.tblh.hw.score, "score")

wald(bmd.tblh.hw.score, "score")$score$anova$"p-value"

wald(bmd.tblh.hw.score, ":score")

wald(bmd.tblh.hw.score, ":score")[[1]]$anova$"p-value"

bmc.tblh.hw.score <- lme(tblh_bmc ~ (I(age-13) + I((age-13)^2) +

I((age-13)^3) + age_g13:I((age-13)^3))*(kz021 + height + weight + score), data, random=~ I(age-13)|ID, method="ML", na.action=na.omit)

summary(bmc.tblh.hw.score)

wald(bmc.tblh.hw.score, "score")

wald(bmc.tblh.hw.score, "score")$score$anova$"p-value"

wald(bmc.tblh.hw.score, ":score")

wald(bmc.tblh.hw.score, ":score")[[1]]$anova$"p-value"

ba.tblh.hw.score <- lme(tblh_ba ~ (I(age-13) + I((age-13)^2) +

I((age-13)^3) + age_g13:I((age-13)^3))*(kz021 + height + weight + score), data, random=~ I(age-13)|ID, method="ML", na.action=na.omit)

summary(ba.tblh.hw.score)

wald(ba.tblh.hw.score, "score")

wald(ba.tblh.hw.score, "score")$score$anova$"p-value"

wald(ba.tblh.hw.score, ":score")

wald(ba.tblh.hw.score, ":score")[[1]]$anova$"p-value"

**Supplementary Figure 1:** Mean (95% CI) BMD at age 9, 13 and 17 years by genetic risk score. The histogram represents the distribution of the genetic risk score. The sample size of each figure is: N=6,896 at age 9, N=5,262 at age 13 and N=3,857 at age 17.

| 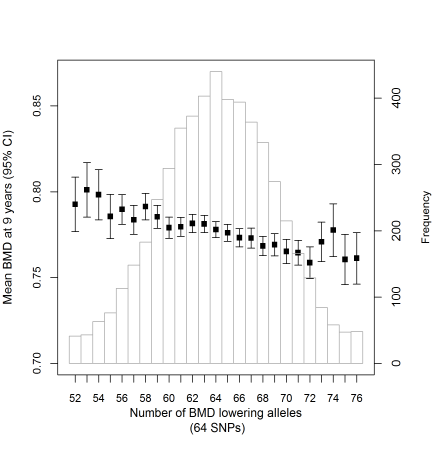 | 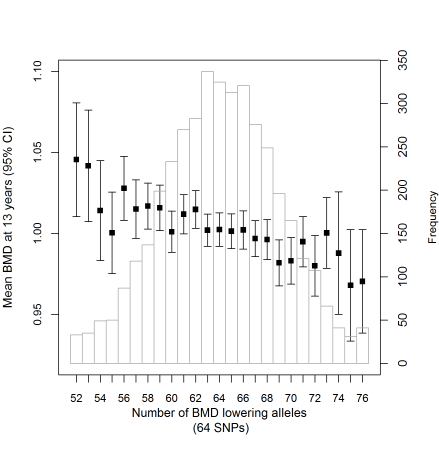 | 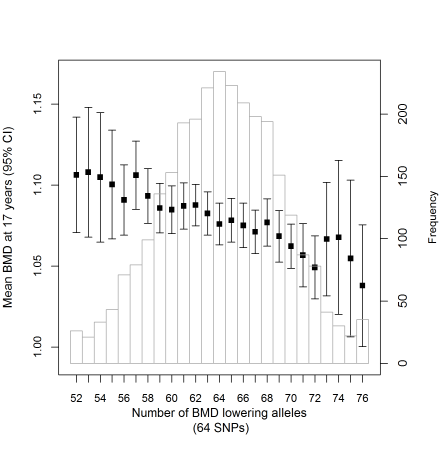 |
| --- | --- | --- |

**Supplementary Figure 2:** The observed BMD measures from each follow-up with the mean predicted trajectories of BMD from age 9 to 17 years for males and females.


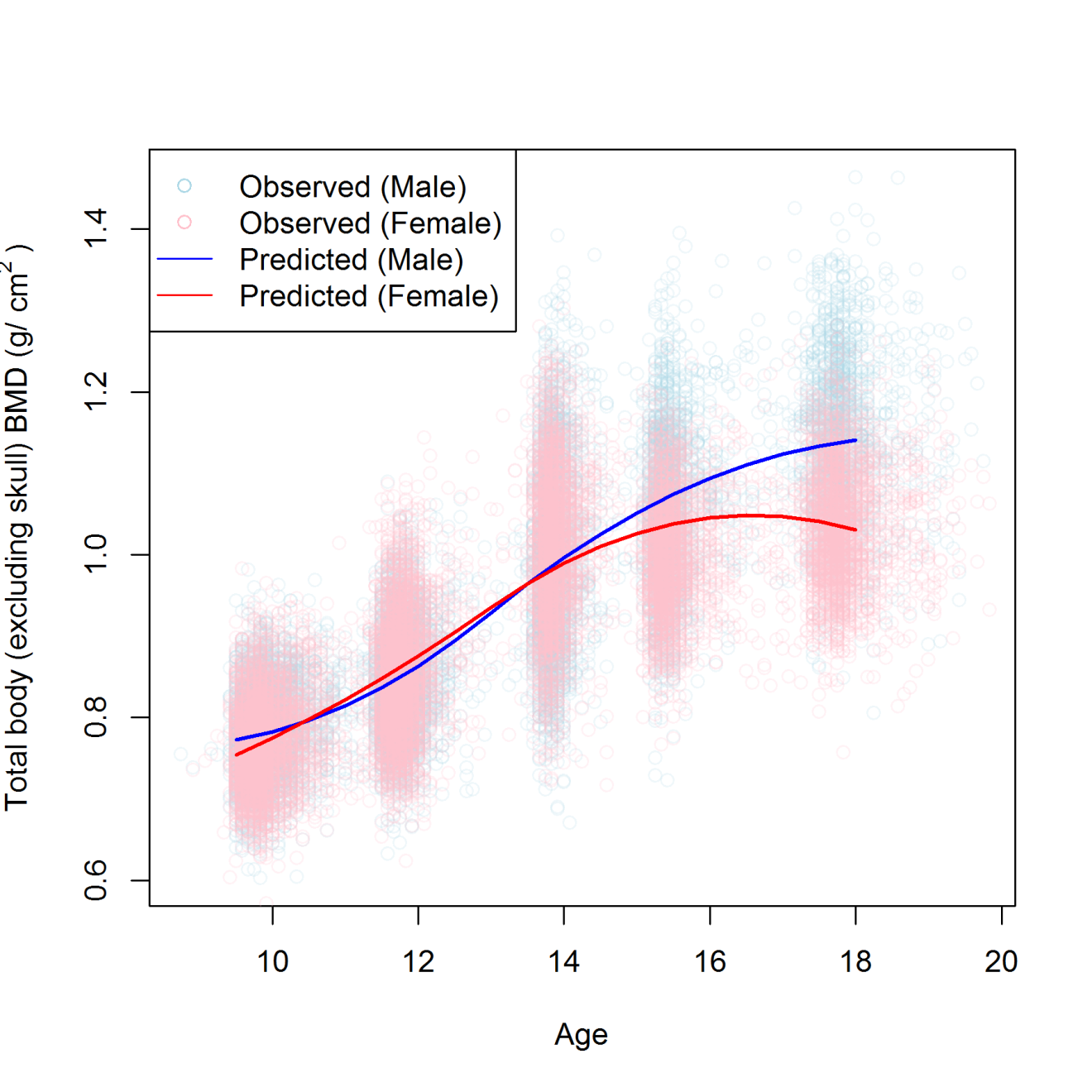


**Supplementary Figure 3:** The observed BA measures from each follow-up with the mean predicted trajectories of BA from age 9 to 17 years for males and females.


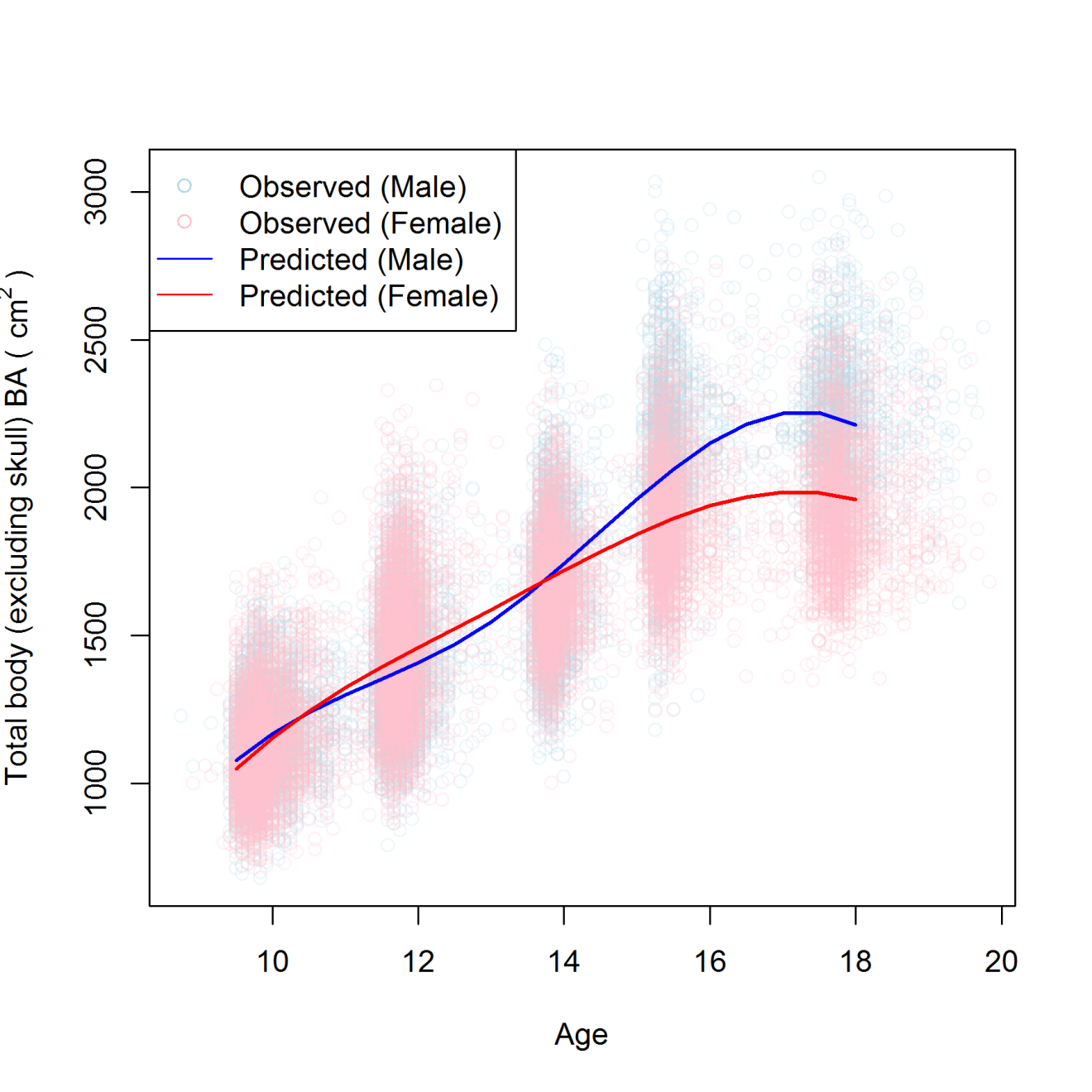


**Supplementary Figure 4:** The observed BMC measures from each follow-up with the mean predicted trajectories of BMC from age 9 to 17 years for males and females.


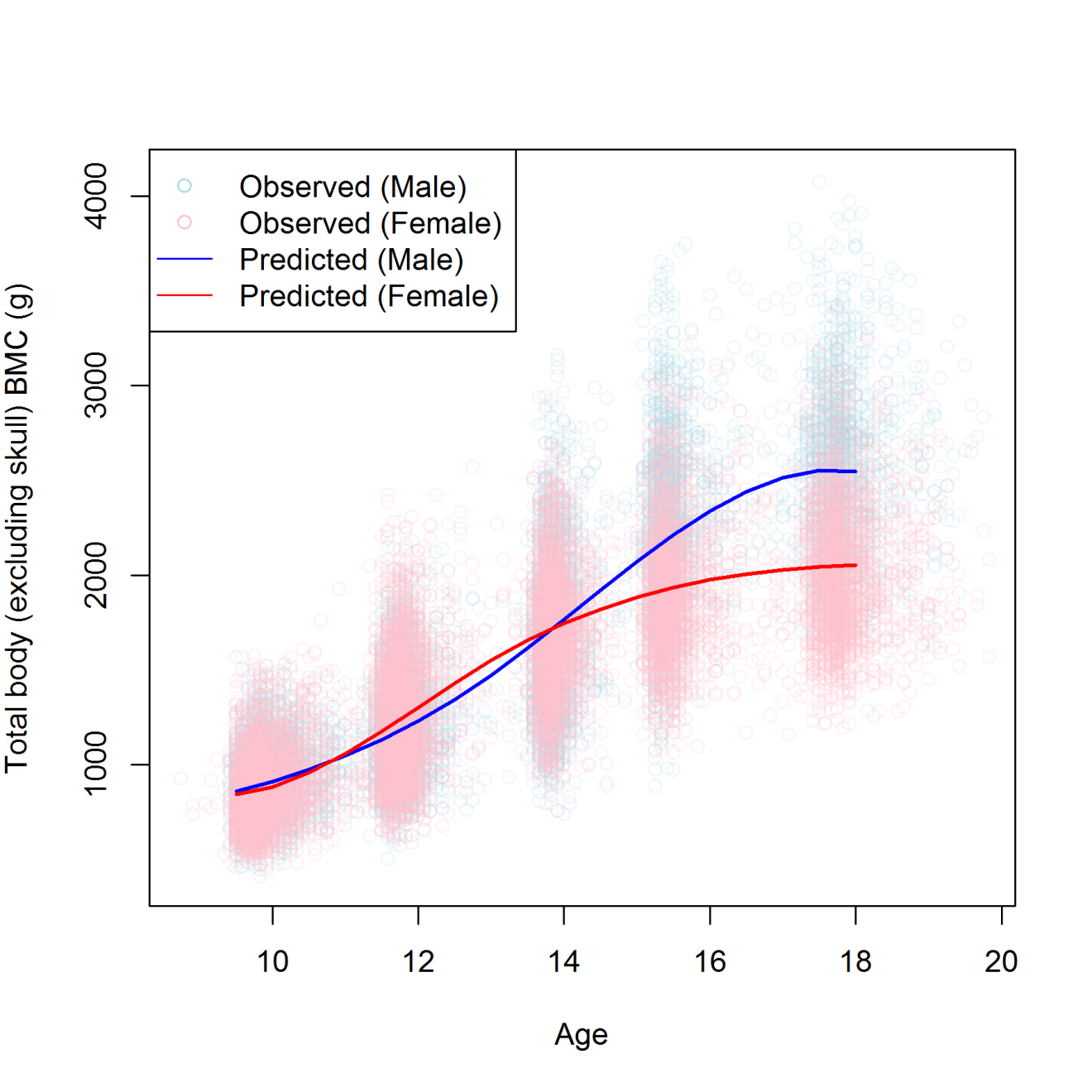


**Supplementary Table 1:** Descriptive statistics of the single nucleotide polymorphisms included in the genetic risk score. The effect allele frequency was calculated from the 6,397 individuals included in the analysis.

| Chromosome | Nearest Gene | SNP | BMD lowering allele/other allele | Effect allele frequency | Imputation quality |
| --- | --- | --- | --- | --- | --- |
| 1 | *DNM3* | rs479336 | T/G | 0.75 | 1.00 |
| 1 | *WLS* | rs17482952 | G/A | 0.08 | 1.00 |
| 1 | *WLS* | rs12407028 | C/T | 0.40 | 0.98 |
| 1 | *WNT4* | rs7521902 | A/C | 0.24 | 1.00 |
| 1 | *ZBTB40* | rs6426749 | G/C | 0.82 | 0.99 |
| 2 | *PKDCC* | rs7584262 | C/T | 0.77 | 0.96 |
| 2 | *ANAPC1* | rs17040773 | C/A | 0.25 | 0.72 |
| 2 | *INSIG2* | rs1878526 | G/A | 0.78 | 0.98 |
| 2 | *SPTBN1* | rs4233949 | G/C | 0.60 | 1.00 |
| 2 | *GALNT3* | rs1346004 | A/G | 0.49 | 0.99 |
| 3 | *KIAA2018* | rs1026364 | G/T | 0.65 | 0.98 |
| 3 | *LEKR1* | rs344081 | C/T | 0.13 | 0.97 |
| 3 | *CTNNB1* | rs430727 | T/C | 0.44 | 0.95 |
| 4 | *IDUA* | rs3755955 | A/G | 0.15 | 1.00 |
| 4 | *MEPE* | rs6532023 | G/T | 0.66 | 1.00 |
| 5 | *MEF2C* | rs1366594 | C/A | 0.46 | 1.00 |
| 6 | *SUPT3H/RUNX2* | rs11755164 | T/C | 0.43 | 0.88 |
| 6 | *CDKAL1/SOX4* | rs9466056 | A/G | 0.38 | 1.00 |
| 6 | *RSPO3* | rs13204965 | C/A | 0.24 | 0.88 |
| 6 | *ESR1* | rs7751941 | A/G | 0.23 | 1.00 |
| 6 | *C6orf97* | rs4869742 | T/C | 0.29 | 1.00 |
| 7 | *WNT16* | rs3801387 | A/G | 0.73 | 1.00 |
| 7 | *C7orf58* | rs13245690 | G/A | 0.38 | 0.99 |
| 7 | *ABCF2* | rs7812088 | G/A | 0.89 | 1.00 |
| 7 | *RXNDC3* | rs10226308 | A/G | 0.82 | 0.99 |
| 7 | *STARD3NL* | rs6959212 | T/C | 0.34 | 0.98 |
| 7 | *SLC25A13* | rs4727338 | G/C | 0.33 | 0.97 |
| 8 | *XKR9/LACTB2* | rs7017914 | G/A | 0.51 | 1.00 |
| 8 | *TNFRSF11B/OPG* | rs2062377 | A/T | 0.57 | 0.99 |
| 9 | *FUBP3* | rs7851693 | G/C | 0.35 | 0.98 |
| 10 | *MPP7* | rs3905706 | C/T | 0.79 | 1.00 |
| 10 | *MBL2/DKK1* | rs1373004 | T/G | 0.12 | 0.88 |
| 10 | *KCNMA1* | rs7071206 | T/C | 0.79 | 1.00 |
| 10 | *CPN1* | rs7084921 | C/T | 0.58 | 0.98 |
| 11 | *LIN7C* | rs10835187 | T/C | 0.55 | 1.00 |
| 11 | *ARHGAP1* | rs7932354 | C/T | 0.70 | 0.99 |
| 11 | *DCDC5* | rs163879 | T/C | 0.68 | 1.00 |
| 11 | *SOX6* | rs7108738 | T/G | 0.83 | 1.00 |
| 11 | *LRP5* | rs3736228 | T/C | 0.16 | 1.00 |
| 12 | *KLHDC5/PTHLH* | rs7953528 | T/A | 0.83 | 0.90 |
| 12 | *ERC1/WNT5B* | rs2887571 | A/G | 0.75 | 0.99 |
| 12 | *DHH* | rs12821008 | C/T | 0.60 | 1.00 |
| 12 | *C12orf23* | rs1053051 | T/C | 0.52 | 1.00 |
| 12 | *HOXC6* | rs736825 | G/C | 0.38 | 0.85 |
| 12 | *SP7* | rs2016266 | A/G | 0.67 | 0.99 |
| 13 | *TNFSF11/RANKL* | rs9533090 | T/C | 0.49 | 1.00 |
| 14 | *RPS6KA5* | rs1286083 | T/C | 0.82 | 1.00 |
| 14 | *MARK3* | rs11623869 | T/G | 0.35 | 1.00 |
| 16 | *NTAN1* | rs4985155 | A/C | 0.67 | 1.00 |
| 16 | *AXIN1* | rs9921222 | T/C | 0.48 | 1.00 |
| 16 | *C16orf38/CLCN7* | rs13336428 | A/G | 0.41 | 1.00 |
| 16 | *SALL1/CYLD* | rs1566045 | T/C | 0.79 | 0.99 |
| 16 | *CYLD* | rs1564981 | A/G | 0.46 | 1.00 |
| 16 | *FOXL1* | rs10048146 | G/A | 0.19 | 1.00 |
| 17 | *SMG6* | rs4790881 | C/A | 0.31 | 1.00 |
| 17 | *SOX9* | rs7217932 | G/A | 0.53 | 0.99 |
| 17 | *SOST* | rs4792909 | G/T | 0.62 | 0.97 |
| 17 | *C17orf53* | rs227584 | A/C | 0.71 | 1.00 |
| 17 | *MAPT* | rs1864325 | T/C | 0.24 | 0.97 |
| 18 | *FAM210A* | rs4796995 | G/A | 0.36 | 1.00 |
| 18 | *TNFRSF11A/RANK* | rs884205 | A/C | 0.29 | 0.92 |
| 19 | *GPATCH1* | rs10416218 | T/C | 0.75 | 1.00 |
| 20 | *JAG1* | rs3790160 | C/T | 0.48 | 0.99 |

**Supplementary Table 2:** Associations between the BMD genetic risk scores (GRS) and BMD from the linear mixed effects model, without adjusting for height and weight.

|  | GRS of 63 SNPs | | Children’s GRS (8 SNPs) | | Fracture GRS (16 SNPs) | | RANK-RANKL-OPG pathway GRS (3 SNPs) | | Mesenchymal Stem Cell Differentiation pathway GRS (3 SNPs) | | WNT signalling pathway GRS (8 SNPs) | |
| --- | --- | --- | --- | --- | --- | --- | --- | --- | --- | --- | --- | --- |
| Effect | Beta | P-Value | Beta | P-Value | Beta | P-Value | Beta | P-Value | Beta | P-Value | Beta | P-Value |
| Score | -0.0020 (0.0002) | 4.30x10^-26^ | -0.0037 (0.0005) | 8.06x10^-13^ | -0.0023 (0.0004) | 4.05x10^-10^ | -0.0010 (0.0008) | 0.223 | -0.0023 (0.0008) | 0.004 | -0.0033 (0.0005) | 2.28x10^-10^ |
| Age:Score | -0.0001 (0.0001) | 0.081 | -0.0002 (0.0002) | 0.436 | -0.00002 (0.0002) | 0.909 | 0.0003 (0.0004) | 0.471 | -0.0005 (0.0004) | 0.205 | -0.0001 (0.0002) | 0.666 |
| Age^2^:Score | -5x10^-6^ (3x10^-5^) | 0.881 | 0.00003 (0.0001) | 0.723 | 0.00004 (0.0001) | 0.556 | 0.0001 (0.0001) | 0.465 | -0.0001 (0.0001) | 0.639 | 0.00005 (0.0001) | 0.605 |
| Age^2^:Score (after age 13) | 4x10^-6^ (5x10^-5^) | 0.933 | 0.00002 (0.0001) | 0.912 | -0.00006 (0.0001) | 0.562 | -0.0002 (0.0002) | 0.397 | 0.0001 (0.0002) | 0.623 | -0.00004 (0.0001) | 0.762 |
| Global Wald test |  | 5.78x10^-27^ |  | 4.08x10^-12^ |  | 2.38x10^-9^ |  | 0.611 |  | 0.024 |  | 4.53x10^-9^ |
| Wald test for score by age interaction |  | 1.65x10^-6^ |  | 0.139 |  | 0.200 |  | 0.808 |  | 0.077 |  | 0.187 |

**Supplementary Table 3:** Associations between the BMD genetic risk scores (GRS) and BMC from the linear mixed effects model, without adjusting for height and weight.

|  | GRS of 63 SNPs | | Children’s GRS (8 SNPs) | | Fracture GRS (16 SNPs) | | RANK-RANKL-OPG pathway GRS (3 SNPs) | | Mesenchymal Stem Cell Differentiation pathway GRS (3 SNPs) | | WNT signalling pathway GRS (8 SNPs) | |
| --- | --- | --- | --- | --- | --- | --- | --- | --- | --- | --- | --- | --- |
| Effect | Beta | P-Value | Beta | P-Value | Beta | P-Value | Beta | P-Value | Beta | P-Value | Beta | P-Value |
| Score | -5.246 (0.788) | 3.13x10^-11^ | -11.060 (2.205) | 5.42x10^-7^ | -5.443 (1.530) | 3.79x10^-4^ | -2.437 (3.354) | 0.468 | -8.413 (3.312) | 0.011 | -9.789 (2.210) | 9.69x10^-6^ |
| Age:Score | -0.648 (0.229) | 0.005 | -1.201 (0.643) | 0.062 | -0.210 (0.447) | 0.638 | 0.512 (0.978) | 0.600 | -2.218 (0.962) | 0.021 | -1.276 (0.646) | 0.048 |
| Age^2^:Score | 0.185 (0.211) | 0.381 | 1.520 (0.604) | 0.012 | 0.282 (0.407) | 0.489 | 1.531 (0.907) | 0.091 | 1.820 (0.882) | 0.039 | 0.548 (0.592) | 0.354 |
| Age^3^:Score | 0.051 (0.071) | 0.475 | 0.419 (0.203) | 0.039 | 0.038 (0.138) | 0.784 | 0.379 (0.306) | 0.215 | 0.633 (0.298) | 0.033 | 0.170 (0.200) | 0.397 |
| Age^3^:Score (after age 13) | -0.091 (0.106) | 0.390 | -0.653 (0.303) | 0.031 | -0.106 (0.205) | 0.604 | -0.678 (0.455) | 0.136 | -0.938 (0.443) | 0.034 | -0.263 (0.297) | 0.376 |
| Global Wald test |  | 8.89x10^-10^ |  | 2.84x10^-6^ |  | 0.009 |  | 0.330 |  | 0.101 |  | 0.001 |
| Wald test for score by age interaction |  | 4.64x10^-6^ |  | 0.005 |  | 0.188 |  | 0.290 |  | 0.082 |  | 0.058 |

**Supplementary Table 4:** Associations between the BMD genetic risk scores (GRS) and BA from the linear mixed effects model, without adjusting for height and weight.

|  | GRS of 63 SNPs | | Children’s GRS (8 SNPs) | | Fracture GRS (16 SNPs) | | RANK-RANKL-OPG pathway GRS (3 SNPs) | | Mesenchymal Stem Cell Differentiation pathway GRS (3 SNPs) | | WNT signalling pathway GRS (8 SNPs) | |
| --- | --- | --- | --- | --- | --- | --- | --- | --- | --- | --- | --- | --- |
| Effect | Beta | P-Value | Beta | P-Value | Beta | P-Value | Beta | P-Value | Beta | P-Value | Beta | P-Value |
| Score | -2.110 (0.552) | 1.33x10^-4^ | -4.986 (1.541) | 0.001 | -1.687 (1.069) | 0.115 | -1.149 (2.343) | 0.624 | -4.013 (2.314) | 0.083 | -3.951 (1.545) | 0.011 |
| Age:Score | 0.061 (0.158) | 0.702 | 0.139 (0.442) | 0.753 | 0.356 (0.308) | 0.248 | 0.238 (0.674) | 0.724 | -0.866 (0.662) | 0.191 | -0.122 (0.445) | 0.784 |
| Age^2^:Score | 0.074 (0.154) | 0.628 | 0.829 (0.440) | 0.060 | -0.019 (0.297) | 0.950 | 1.420 (0.660) | 0.032 | 1.003 (0.642) | 0.118 | 0.061 (0.431) | 0.888 |
| Age^3^:Score | 0.005 (0.052) | 0.927 | 0.180 (0.149) | 0.226 | -0.041 (0.102) | 0.686 | 0.374 (0.224) | 0.095 | 0.368 (0.218) | 0.092 | 0.015 (0.147) | 0.919 |
| Age^3^:Score (after age 13) | -0.033 (0.077) | 0.674 | -0.334 (0.222) | 0.132 | 0.021 (0.150) | 0.887 | -0.646 (0.333) | 0.052 | -0.533 (0.323) | 0.099 | -0.048 (0.217) | 0.825 |
| Global Wald test |  | 2.79x10^-4^ |  | 0.007 |  | 0.200 |  | 0.196 |  | 0.433 |  | 0.044 |
| Wald test for score by age interaction |  | 0.056 |  | 0.097 |  | 0.475 |  | 0.118 |  | 0.561 |  | 0.285 |

**Supplementary Table 5:** Associations between the BMD genetic risk scores and BMD at each follow-up from the univariate linear model adjusting for age, sex, height and weight.

|  | Year 9 | Year 11 | Year 13 | Year 15 | Year 17 |
| --- | --- | --- | --- | --- | --- |
| Genetic risk score of 63 SNPs | -0.0015 (0.0001), P=4.60x10^-48^ [r^2^=0.020] | -0.0017 (0.0001), P=4.38x10^-41^ [r^2^=0.016] | -0.0021 (0.0002), P=1.05x10^-23^ [r^2^=0.011] | -0.0022 (0.0002), P=1.88x10^-21^ [r^2^=0.016] | -0.0026 (0.0003), P=2.79x10^-24^ [r^2^=0.017] |
| Children’s genetic risk score (8 SNPs) | -0.0028 (0.0003), P=1.88x10^-21^ [r^2^=0.009] | -0.0029 (0.0004), P=6.42x10^-16^ [r^2^=0.006] | -0.0031 (0.0006), P=1.01x10^-7^ [r^2^=0.003] | -0.0030 (0.0007), P=3.78x10^-6^ [r^2^=0.004] | -0.0035 (0.0007), P=1.55x10^-6^ [r^2^=0.004] |
| Fracture genetic risk score (16 SNPs) | -0.0020 (0.0002), P=3.08x10^-22^ [r^2^=0.009] | -0.0022 (0.0002), P=2.98x10^-18^ [r^2^=0.007] | -0.0029 (0.0004), P=2.71x10^-12^ [r^2^=0.005] | -0.0027 (0.0005), P=1.28x10^-9^ [r^2^=0.006] | -0.0033 (0.0005), P=4.44x10^-11^ [r^2^=0.007] |
| RANK-RANKL-OPG pathway genetic risk score (3 SNPs) | -0.0011 (0.0005), P=0.01 [r^2^=0.0005] | -0.0014 (0.0005), P=0.01 [r^2^=0.0005] | -0.0008 (0.0009), P=0.37 [r^2^=0.0000] | -0.0006 (0.0010),  P=0.54 [r^2^=0.0000] | -0.0018 (0.0011),  P=0.11 [r^2^=0.0003] |
| Mesenchymal Stem Cell Differentiation pathway genetic risk score (3 SNPs) | -0.0012 (0.0004), P=9.33x10^-3^ [r^2^=0.0006] | -0.0016 (0.0005), P=2.28x10^-3^ [r^2^=0.0008] | -0.0028 (0.0009), P=1.32x10^-3^ [r^2^=0.0010] | -0.0036 (0.0010), P=3.36x10^-4^ [r^2^=0.0021] | -0.0031 (0.0011),  P=3.88x10^-3^ [r^2^=0.0013] |
| WNT signalling pathway genetic risk score (8 SNPs) | -0.0024 (0.0003), P=9.07x10^-16^ [r^2^=0.006] | -0.0026 (0.0004), P=2.57x10^-13^ [r^2^=0.005] | -0.0032 (0.0006), P=4.83x10^-8^ [r^2^=0.003] | -0.0033 (0.0007), P=4.14x10^-7^ [r^2^=0.004] | -0.0040 (0.0007), P=4.25x10^-8^ [r^2^=0.005] |

**Supplementary Table 6:** Associations between the BMD genetic risk scores and BMC at each follow-up from the univariate linear model adjusting for age, sex, height and weight.

|  | Year 9 | Year 11 | Year 13 | Year 15 | Year 17 |
| --- | --- | --- | --- | --- | --- |
| Genetic risk score of 63 SNPs | -2.749 (0.2217), P=8.33x10^-35^ [r^2^=0.006] | -3.896 (0.3295), P=7.71x10^-32^ [r^2^=0.005] | -5.109 (0.5248), P=3.70x10^-22^ [r^2^=0.005] | -6.614 (0.7359), P=4.33x10^-19^ [r^2^=0.006] | -7.730 (0.7889), P=2.56x10^-22^ [r^2^=0.007] |
| Children’s genetic risk score (8 SNPs) | -5.216 (0.6216), P=6.10x10^-17^ [r^2^=0.003] | -6.236 (0.9254), P=1.77x10^-11^ [r^2^=0.002] | -7.758 (1.4842), P=1.81x10^-7^ [r^2^=0.002] | -9.654 (2.090), P=3.99x10^-6^ [r^2^=0.002] | -9.992 (2.3051), P=1.51x10^-5^ [r^2^=0.001] |
| Fracture genetic risk score (16 SNPs) | -3.552 (0.4313), P=2.22x10^-16^ [r^2^=0.003] | -5.013 (0.6506), P=1.56x10^-14^ [r^2^=0.002] | -6.648 (1.029),  P=1.17x10^-10^ [r^2^=0.002] | -8.459 (1.4352), P=4.17x10^-9^ [r^2^=0.003] | -10.992 (1.5633), P=2.55x10^-12^ [r^2^=0.004] |
| RANK-RANKL-OPG pathway genetic risk score (3 SNPs) | -2.435 (0.9559), P=0.01 [r^2^=0.0002] | -3.553 (1.4251), P=0.01 [r^2^=0.0002] | -1.249 (2.2611), P=0.58 [r^2^=0.0000] | -1.492 (3.1669), P=0.64 [r^2^=0.0000] | -4.596 (3.468), P=0.19 [r^2^=0.0000] |
| Mesenchymal Stem Cell Differentiation pathway genetic risk score (3 SNPs) | -2.194 (0.9386), P=0.02 [r^2^=0.0002] | -2.851 (1.3967), P=0.04 [r^2^=0.0001] | -6.704 (2.2214), P=2.56x10^-3^ [r^2^=0.0005] | -7.266 (3.1555), P=0.02 [r^2^=0.0003] | -8.173 (3.3776), P=0.02 [r^2^=0.0004] |
| WNT signalling pathway genetic risk score (8 SNPs) | -4.671 (0.6310), P=1.54x10^-13^ [r^2^=0.002] | -5.734 (0.9367), P=9.94x10^-10^ [r^2^=0.001] | -9.122 (1.4762), P=7.07x10^-10^ [r^2^=0.002] | -11.492 (2.0933), P=4.35x10^-8^ [r^2^=0.002] | -12.462 (2.2705), P=4.40x10^-8^ [r^2^=0.002] |

**Supplementary Table 7:** Associations between the BMD genetic risk scores and BA at each follow-up from the univariate linear model adjusting for age, sex, height and weight.

|  | Year 9 | Year 11 | Year 13 | Year 15 | Year 17 |
| --- | --- | --- | --- | --- | --- |
| Genetic risk score of 63 SNPs | -1.319 (0.1721), P=2.18x10^-14^ [r^2^=0.0016] | -1.760 (0.2225), P=3.12x10^-15^ [r^2^=0.0016] | -1.582 (0.2578), P=9.27x10^-10^ [r^2^=0.0015] | -2.199 (0.3755), P=5.25x10^-9^ [r^2^=0.0017] | -2.463 (0.3928), P=4.15x10^-10^ [r^2^=0.0022] |
| Children’s genetic risk score (8 SNPs) | -2.719 (0.4799), P=1.55x10^-8^ [r^2^=0.0009] | -2.868 (0.6216), P=4.05x10^-6^ [r^2^=0.0005] | -2.522 (0.7256), P=5.14x10^-4^ [r^2^=0.0005] | -3.961 (1.0595), P=1.89x10^-4^ [r^2^=0.0007] | -2.518 (1.1395), P=0.03 [r^2^=0.0002] |
| Fracture genetic risk score (16 SNPs) | -1.717 (0.3309), P=2.63x10^-7^ [r^2^=0.0007] | -2.202 (0.4375), P=4.97x10^-7^ [r^2^=0.0006] | -1.815 (0.5039), P=3.19x10^-4^ [r^2^=0.0005] | -2.945 (0.7290), P=5.50x10^-5^ [r^2^=0.0008] | -4.223 (0.7735), P=5.17x10^-8^ [r^2^=0.0016] |
| RANK-RANKL-OPG pathway genetic risk score (3 SNPs) | -1.563 (0.7355), P=0.03 [r^2^=0.0000] | -1.874 (0.9553), P=0.05 [r^2^=0.0000] | -0.207 (1.1033), P=0.85 [r^2^=0.0000] | -0.926 (1.6038), P=0.56 [r^2^=0.0000] | -0.788 (1.7106), P=0.64 [r^2^=0.0000] |
| Mesenchymal Stem Cell Differentiation pathway genetic risk score (3 SNPs) | -1.159 (0.7223), P=0.11 [r^2^=0.0000] | -1.022 (0.9363), P=0.28 [r^2^=0.0000] | -2.167 (1.0846), P=0.05 [r^2^=0.0001] | -0.878 (1.5994), P=0.58 [r^2^=0.0000] | -2.387 (1.6668), P=0.15 [r^2^=0.0000] |
| WNT signalling pathway genetic risk score (8 SNPs) | -2.471 (0.4868), P=3.98x10^-7^ [r^2^=0.0007] | -2.149 (0.6293), P=6.45x10^-4^ [r^2^=0.0003] | -3.462 (0.7216), P=1.66x10^-6^ [r^2^=0.0009] | -4.513 (1.0623), P=2.22x10^-5^ [r^2^=0.0009] | -4.282 (1.1227), P=1.39x10^-4^ [r^2^=0.0008] |

**Supplementary Table 8:** Association results for the 63 SNPs and BMD acquisition. Coefficients (standard error) represent the mean difference in BMD (g/cm^2^) or growth (g/cm^2^) per year per ‘BMD lowering’ allele.

| SNP | Beta SNP | SE SNP | P SNP | Beta SNP:age | SE SNP:age | P SNP:age | Beta SNP:age^2^ | SE SNP:age^2^ | P SNP:age^2^ | Beta SNP:age^2^ (after 13 years) | SE SNP:age^2^ (after 13 years) | P SNP:age^2^ (after 13 years) | Global Wald | Wald test for SNP by age interaction |
| --- | --- | --- | --- | --- | --- | --- | --- | --- | --- | --- | --- | --- | --- | --- |
| rs7521902 | -0.0040 | 0.0016 | 0.0113 | -0.0009 | 0.0007 | 0.2086 | -0.0001 | 0.0003 | 0.7190 | 0.0003 | 0.0004 | 0.4156 | 0.0769 | 0.2291 |
| rs6426749 | -0.0059 | 0.0017 | 0.0007 | -0.0001 | 0.0008 | 0.8692 | 0.0002 | 0.0003 | 0.5191 | -0.0002 | 0.0004 | 0.6840 | 0.0190 | 0.3840 |
| rs17482952 | -0.0045 | 0.0024 | 0.0657 | 0.0012 | 0.0011 | 0.2974 | 0.0006 | 0.0004 | 0.1787 | -0.0006 | 0.0006 | 0.3460 | 0.0464 | 0.1734 |
| rs12407028 | -0.0030 | 0.0014 | 0.0256 | 2.03E-05 | 0.0006 | 0.9734 | 0.0001 | 0.0002 | 0.7375 | -0.0002 | 0.0003 | 0.6445 | 0.1663 | 0.4901 |
| rs479336 | -0.0028 | 0.0015 | 0.0680 | -0.0007 | 0.0007 | 0.2846 | -0.0002 | 0.0003 | 0.5003 | 0.0004 | 0.0004 | 0.3143 | 0.1835 | 0.5090 |
| rs7584262 | -0.0035 | 0.0016 | 0.0283 | -0.0005 | 0.0007 | 0.4520 | 6.71E-06 | 0.0003 | 0.9804 | 0.0001 | 0.0004 | 0.8131 | 0.2649 | 0.4289 |
| rs4233949 | -0.0030 | 0.0013 | 0.0249 | -0.0003 | 0.0006 | 0.6351 | 4.21E-05 | 0.0002 | 0.8545 | -0.0001 | 0.0003 | 0.8149 | 0.1245 | 0.1585 |
| rs17040773 | -0.0004 | 0.0018 | 0.8438 | -2.50E-05 | 0.0008 | 0.9758 | 0.0000 | 0.0003 | 0.9832 | 0.0001 | 0.0005 | 0.9056 | 0.9620 | 0.9202 |
| rs1878526 | 0.0011 | 0.0016 | 0.5114 | 0.0008 | 0.0007 | 0.2822 | -4.61E-05 | 0.0003 | 0.8688 | -0.0001 | 0.0004 | 0.7376 | 0.1053 | 0.0818 |
| rs1346004 | -0.0042 | 0.0013 | 0.0017 | 0.0013 | 0.0006 | 0.0336 | 0.0004 | 0.0002 | 0.0702 | -0.0007 | 0.0003 | 0.0447 | 0.0002 | 0.2042 |
| rs430727 | -0.0011 | 0.0014 | 0.4048 | 0.0002 | 0.0006 | 0.7196 | 0.0001 | 0.0002 | 0.6083 | -0.0003 | 0.0003 | 0.4483 | 0.4843 | 0.3461 |
| rs1026364 | -0.0026 | 0.0014 | 0.0655 | -0.0018 | 0.0006 | 0.0056 | -0.0004 | 0.0002 | 0.0675 | 0.0007 | 0.0004 | 0.0478 | 0.0159 | 0.0085 |
| rs344081 | 0.0009 | 0.0020 | 0.6600 | 0.0010 | 0.0009 | 0.2688 | 0.0004 | 0.0003 | 0.3034 | -0.0007 | 0.0005 | 0.1717 | 0.3006 | 0.2521 |
| rs3755955 | -0.0025 | 0.0018 | 0.1791 | -0.0011 | 0.0008 | 0.1766 | -0.0004 | 0.0003 | 0.2361 | 0.0005 | 0.0005 | 0.2519 | 0.3772 | 0.5065 |
| rs6532023 | -0.0019 | 0.0014 | 0.1626 | 0.0005 | 0.0006 | 0.4445 | 0.0001 | 0.0002 | 0.6178 | -0.0002 | 0.0004 | 0.5173 | 0.2936 | 0.8590 |
| rs1366594 | -0.0024 | 0.0013 | 0.0681 | 0.0003 | 0.0006 | 0.6673 | 0.0001 | 0.0002 | 0.6702 | -0.0002 | 0.0003 | 0.4755 | 0.1752 | 0.4236 |
| rs9466056 | -0.0017 | 0.0013 | 0.2020 | -0.0004 | 0.0006 | 0.4828 | -0.0001 | 0.0002 | 0.5612 | 0.0002 | 0.0003 | 0.5543 | 0.6352 | 0.9021 |
| rs11755164 | -0.0018 | 0.0014 | 0.2045 | 2.57E-05 | 0.0006 | 0.9680 | 4.81E-05 | 0.0002 | 0.8425 | -0.0001 | 0.0004 | 0.8049 | 0.7418 | 0.9132 |
| rs13204965 | -0.0064 | 0.0016 | 0.0001 | 0.0009 | 0.0007 | 0.2069 | 0.0005 | 0.0003 | 0.0782 | -0.0006 | 0.0004 | 0.1470 | 0.0002 | 0.2537 |
| rs4869742 | -0.0018 | 0.0015 | 0.2163 | -0.0014 | 0.0007 | 0.0294 | -0.0003 | 0.0002 | 0.2733 | 0.0005 | 0.0004 | 0.2076 | 0.0277 | 0.0129 |
| rs7751941 | 0.0003 | 0.0016 | 0.8242 | -0.0012 | 0.0007 | 0.0843 | -0.0003 | 0.0003 | 0.3333 | 0.0004 | 0.0004 | 0.3043 | 0.0488 | 0.0796 |
| rs10226308 | -0.0034 | 0.0017 | 0.0461 | -0.0002 | 0.0008 | 0.7809 | 3.55E-05 | 0.0003 | 0.9035 | -1.37E-05 | 0.0004 | 0.9752 | 0.3822 | 0.8355 |
| rs6959212 | -0.0028 | 0.0014 | 0.0420 | 0.0006 | 0.0006 | 0.3566 | 0.0002 | 0.0002 | 0.3211 | -0.0003 | 0.0004 | 0.4214 | 0.0656 | 0.5753 |
| rs4727338 | -0.0035 | 0.0014 | 0.0125 | 2.32E-05 | 0.0006 | 0.9713 | 0.0001 | 0.0002 | 0.7849 | -0.0001 | 0.0004 | 0.8193 | 0.1044 | 0.9512 |
| rs13245690 | -0.0030 | 0.0014 | 0.0266 | -0.0008 | 0.0006 | 0.1995 | -0.0002 | 0.0002 | 0.3974 | 0.0004 | 0.0003 | 0.2591 | 0.0982 | 0.5141 |
| rs3801387 | -0.0043 | 0.0015 | 0.0037 | -0.0002 | 0.0007 | 0.8005 | -4.63E-05 | 0.0003 | 0.8562 | 4.42E-05 | 0.0004 | 0.9087 | 0.0161 | 0.9416 |
| rs7812088 | -0.0020 | 0.0021 | 0.3245 | -0.0010 | 0.0009 | 0.2933 | -0.0005 | 0.0004 | 0.1795 | 0.0007 | 0.0005 | 0.2175 | 0.2879 | 0.5965 |
| rs7017914 | -0.0023 | 0.0013 | 0.0880 | -0.0005 | 0.0006 | 0.4117 | -0.0001 | 0.0002 | 0.8080 | 0.0002 | 0.0003 | 0.4747 | 0.1781 | 0.3120 |
| rs2062377 | -0.0008 | 0.0014 | 0.5757 | -0.0005 | 0.0006 | 0.4465 | -0.0001 | 0.0002 | 0.6563 | 0.0001 | 0.0003 | 0.6691 | 0.8081 | 0.6595 |
| rs7851693 | -0.0034 | 0.0014 | 0.0167 | -0.0003 | 0.0006 | 0.6057 | -0.0001 | 0.0002 | 0.7157 | 0.0001 | 0.0004 | 0.8113 | 0.0884 | 0.6729 |
| rs3905706 | -0.0001 | 0.0016 | 0.9442 | -0.0003 | 0.0007 | 0.7318 | 0.0000 | 0.0003 | 0.9895 | 0.0001 | 0.0004 | 0.9006 | 0.9519 | 0.9155 |
| rs1373004 | -0.0015 | 0.0021 | 0.4856 | 0.0002 | 0.0009 | 0.7944 | 0.0001 | 0.0004 | 0.8337 | -0.0001 | 0.0005 | 0.8773 | 0.8504 | 0.9647 |
| rs7071206 | -0.0027 | 0.0016 | 0.0956 | -0.0001 | 0.0007 | 0.8994 | 0.0001 | 0.0003 | 0.7798 | -2.61E-05 | 0.0004 | 0.9497 | 0.5179 | 0.8481 |
| rs7084921 | 0.0001 | 0.0014 | 0.9128 | 0.0008 | 0.0006 | 0.1883 | 0.0002 | 0.0002 | 0.4556 | -0.0004 | 0.0003 | 0.2704 | 0.5323 | 0.4032 |
| rs7108738 | -0.0041 | 0.0017 | 0.0182 | -0.0009 | 0.0008 | 0.2711 | -0.0002 | 0.0003 | 0.5875 | 0.0003 | 0.0004 | 0.4641 | 0.1538 | 0.5533 |
| rs10835187 | -0.0043 | 0.0013 | 0.0010 | -0.0014 | 0.0006 | 0.0166 | -0.0003 | 0.0002 | 0.2054 | 0.0005 | 0.0003 | 0.1075 | 0.0040 | 0.0231 |
| rs163879 | 0.0028 | 0.0014 | 0.0442 | -0.0001 | 0.0006 | 0.8191 | -0.0002 | 0.0002 | 0.4982 | 0.0001 | 0.0004 | 0.7275 | 0.1959 | 0.5977 |
| rs7932354 | -0.0040 | 0.0014 | 0.0057 | 0.0002 | 0.0007 | 0.7543 | 0.0002 | 0.0002 | 0.3354 | -0.0003 | 0.0004 | 0.3648 | 0.0806 | 0.3202 |
| rs3736228 | -0.0049 | 0.0018 | 0.0073 | -0.0005 | 0.0008 | 0.5873 | 0.0001 | 0.0003 | 0.6914 | 0.0000 | 0.0005 | 0.9967 | 0.0992 | 0.2880 |
| rs2887571 | -0.0027 | 0.0015 | 0.0775 | 0.0004 | 0.0007 | 0.5720 | 0.0001 | 0.0003 | 0.6854 | -0.0002 | 0.0004 | 0.5521 | 0.2016 | 0.8430 |
| rs7953528 | -0.0006 | 0.0018 | 0.7490 | -0.0004 | 0.0008 | 0.5846 | -0.0002 | 0.0003 | 0.5110 | 0.0003 | 0.0005 | 0.4846 | 0.8858 | 0.8883 |
| rs12821008 | -0.0016 | 0.0013 | 0.2439 | -4.03E-05 | 0.0006 | 0.9469 | 1.97E-05 | 0.0002 | 0.9313 | -4.69E-05 | 0.0003 | 0.8913 | 0.7920 | 0.9168 |
| rs2016266 | -0.0049 | 0.0014 | 0.0005 | -0.0006 | 0.0006 | 0.3393 | -2.99E-05 | 0.0002 | 0.9003 | 0.0001 | 0.0004 | 0.8129 | 0.0095 | 0.1724 |
| rs736825 | 0.0014 | 0.0015 | 0.3569 | -0.0004 | 0.0007 | 0.5477 | -0.0002 | 0.0003 | 0.4005 | 0.0002 | 0.0004 | 0.6372 | 0.3092 | 0.3220 |
| rs1053051 | -0.0021 | 0.0013 | 0.1070 | -4.23E-05 | 0.0006 | 0.9428 | 0.0000 | 0.0002 | 0.9868 | 3.12E-05 | 0.0003 | 0.9257 | 0.3771 | 0.9746 |
| rs9533090 | -0.0006 | 0.0013 | 0.6636 | 0.0007 | 0.0006 | 0.2485 | 0.0002 | 0.0002 | 0.4324 | -0.0004 | 0.0003 | 0.2718 | 0.5555 | 0.5030 |
| rs1286083 | -0.0042 | 0.0017 | 0.0129 | -0.0005 | 0.0008 | 0.4867 | -0.0002 | 0.0003 | 0.5725 | 0.0004 | 0.0004 | 0.3081 | 0.0013 | 0.1344 |
| rs11623869 | 0.0005 | 0.0014 | 0.7131 | 0.0004 | 0.0006 | 0.5143 | 0.0002 | 0.0002 | 0.5014 | -0.0004 | 0.0004 | 0.3016 | 0.2091 | 0.1875 |
| rs9921222 | -0.0055 | 0.0013 | 2.10E-05 | -0.0011 | 0.0006 | 0.0734 | -0.0002 | 0.0002 | 0.4749 | 0.0004 | 0.0003 | 0.1855 | 0.0001 | 0.0601 |
| rs13336428 | -0.0003 | 0.0014 | 0.8530 | 0.0010 | 0.0006 | 0.0986 | 0.0002 | 0.0002 | 0.3327 | -0.0005 | 0.0003 | 0.1662 | 0.2603 | 0.2309 |
| rs4985155 | 0.0026 | 0.0014 | 0.0655 | 0.0001 | 0.0006 | 0.8411 | -1.22E-05 | 0.0002 | 0.9597 | -2.22E-05 | 0.0004 | 0.9511 | 0.3895 | 0.9641 |
| rs1564981 | -0.0003 | 0.0013 | 0.8393 | 0.0003 | 0.0006 | 0.6014 | 0.0001 | 0.0002 | 0.7220 | -0.0002 | 0.0003 | 0.5428 | 0.8334 | 0.7026 |
| rs1566045 | -0.0029 | 0.0016 | 0.0762 | -0.0007 | 0.0008 | 0.3692 | -0.0001 | 0.0003 | 0.7937 | 0.0001 | 0.0004 | 0.7676 | 0.2991 | 0.2770 |
| rs10048146 | -0.0019 | 0.0017 | 0.2638 | 0.0001 | 0.0008 | 0.9024 | 0.0001 | 0.0003 | 0.7279 | -0.0002 | 0.0004 | 0.7093 | 0.8258 | 0.8727 |
| rs4790881 | -0.0033 | 0.0014 | 0.0200 | -0.0009 | 0.0006 | 0.1580 | -0.0001 | 0.0002 | 0.5753 | 0.0003 | 0.0004 | 0.3869 | 0.1382 | 0.2416 |
| rs4792909 | -0.0027 | 0.0014 | 0.0497 | -0.0006 | 0.0006 | 0.3590 | -0.0001 | 0.0002 | 0.6560 | 0.0001 | 0.0004 | 0.7829 | 0.1021 | 0.1114 |
| rs227584 | -0.0003 | 0.0015 | 0.8242 | 0.0004 | 0.0007 | 0.5775 | 3.93E-05 | 0.0002 | 0.8748 | -0.0002 | 0.0004 | 0.6803 | 0.8116 | 0.7857 |
| rs1864325 | -0.0002 | 0.0016 | 0.8731 | -0.0002 | 0.0007 | 0.7675 | -0.0001 | 0.0003 | 0.6789 | 4.47E-05 | 0.0004 | 0.9112 | 0.6581 | 0.4895 |
| rs7217932 | -0.0005 | 0.0013 | 0.7140 | -0.0007 | 0.0006 | 0.2205 | -0.0002 | 0.0002 | 0.4139 | 0.0003 | 0.0003 | 0.4289 | 0.5038 | 0.3601 |
| rs4796995 | 0.0001 | 0.0014 | 0.9439 | 0.0008 | 0.0006 | 0.1873 | 0.0002 | 0.0002 | 0.3047 | -0.0004 | 0.0004 | 0.2424 | 0.7564 | 0.6088 |
| rs884205 | -0.0019 | 0.0015 | 0.2279 | 0.0006 | 0.0007 | 0.3610 | 0.0003 | 0.0003 | 0.3146 | -0.0003 | 0.0004 | 0.3992 | 0.4186 | 0.6461 |
| rs10416218 | 0.0015 | 0.0015 | 0.3433 | -0.0012 | 0.0007 | 0.1015 | -0.0005 | 0.0003 | 0.0430 | 0.0006 | 0.0004 | 0.1489 | 0.0158 | 0.0115 |
| rs3790160 | -0.0004 | 0.0013 | 0.7896 | 0.0005 | 0.0006 | 0.3759 | 0.0002 | 0.0002 | 0.4891 | -0.0004 | 0.0003 | 0.2906 | 0.4889 | 0.3343 |

**Supplementary Table 9:** Association results for the 63 SNPs and BMC acquisition. Coefficients (standard error) represent the mean difference in BMC (g) or growth (g) per year per ‘BMD lowering’ allele.

| SNP | Beta SNP | SE SNP | P SNP | Beta SNP:age | SE SNP:age | P SNP:age | Beta SNP:age^2^ | SE SNP:age^2^ | P SNP:age^2^ | Beta SNP:age^3^ | SE SNP:age^3^ | P SNP:age^3^ | Beta SNP:age^3^ (after 13 years) | SE SNP:age^3^ (after 13 years) | P SNP:age^3^ (after 13 years) | Global Wald | Wald for SNP by age interaction |
| --- | --- | --- | --- | --- | --- | --- | --- | --- | --- | --- | --- | --- | --- | --- | --- | --- | --- |
| rs10416218 | 11.065 | 6.445 | 0.086 | -1.838 | 1.883 | 0.329 | -1.035 | 1.746 | 0.553 | 0.108 | 0.589 | 0.855 | 0.002 | 0.877 | 0.998 | 0.001 | 0.003 |
| rs17482952 | -21.885 | 10.201 | 0.032 | 0.012 | 3.008 | 0.997 | 3.380 | 2.742 | 0.218 | 0.606 | 0.932 | 0.515 | -1.023 | 1.380 | 0.459 | 0.001 | 0.006 |
| rs1026364 | -9.078 | 5.861 | 0.121 | -6.086 | 1.698 | 3.38E-04 | 1.483 | 1.574 | 0.346 | 0.864 | 0.529 | 0.102 | -0.958 | 0.788 | 0.224 | 0.019 | 0.009 |
| rs4790881 | -10.878 | 5.984 | 0.069 | -3.439 | 1.731 | 0.047 | -2.159 | 1.586 | 0.174 | -0.528 | 0.535 | 0.324 | 1.061 | 0.795 | 0.182 | 0.016 | 0.011 |
| rs2887571 | -12.563 | 6.478 | 0.053 | -0.925 | 1.907 | 0.628 | 3.468 | 1.709 | 0.042 | 1.026 | 0.581 | 0.077 | -1.787 | 0.858 | 0.037 | 0.020 | 0.018 |
| rs9921222 | -18.585 | 5.446 | 0.001 | -4.358 | 1.582 | 0.006 | 1.825 | 1.465 | 0.213 | 0.665 | 0.494 | 0.178 | -0.845 | 0.736 | 0.251 | 0.006 | 0.018 |
| rs10835187 | -19.150 | 5.530 | 0.001 | -4.503 | 1.604 | 0.005 | 1.353 | 1.470 | 0.357 | 0.544 | 0.499 | 0.276 | -0.671 | 0.739 | 0.364 | 0.011 | 0.028 |
| rs13336428 | 3.604 | 5.701 | 0.527 | 3.811 | 1.654 | 0.021 | -2.055 | 1.518 | 0.176 | -0.844 | 0.513 | 0.100 | 1.018 | 0.761 | 0.181 | 0.055 | 0.030 |
| rs1566045 | -4.498 | 6.926 | 0.516 | -0.269 | 2.017 | 0.894 | -2.444 | 1.879 | 0.193 | -0.731 | 0.633 | 0.249 | 1.046 | 0.944 | 0.268 | 0.073 | 0.039 |
| rs2016266 | -22.004 | 5.845 | 1.68E-04 | -3.922 | 1.691 | 0.020 | 3.503 | 1.552 | 0.024 | 1.102 | 0.524 | 0.036 | -1.671 | 0.779 | 0.032 | 0.009 | 0.040 |
| rs1878526 | 7.341 | 6.789 | 0.280 | 4.456 | 1.976 | 0.024 | -1.111 | 1.802 | 0.537 | -0.446 | 0.609 | 0.464 | 0.462 | 0.904 | 0.609 | 0.066 | 0.044 |
| rs7751941 | 2.535 | 6.601 | 0.701 | -3.047 | 1.913 | 0.111 | -2.680 | 1.753 | 0.126 | -0.529 | 0.592 | 0.372 | 1.171 | 0.879 | 0.183 | 0.062 | 0.047 |
| rs736825 | 6.892 | 6.149 | 0.262 | -1.489 | 1.788 | 0.405 | -1.398 | 1.612 | 0.386 | -0.154 | 0.547 | 0.778 | 0.357 | 0.810 | 0.660 | 0.025 | 0.052 |
| rs4792909 | -9.709 | 5.824 | 0.096 | -2.553 | 1.696 | 0.132 | 1.696 | 1.546 | 0.273 | 0.617 | 0.523 | 0.238 | -0.973 | 0.776 | 0.210 | 0.133 | 0.078 |
| rs884205 | -4.153 | 6.457 | 0.520 | 1.567 | 1.877 | 0.404 | 2.524 | 1.748 | 0.149 | 0.506 | 0.588 | 0.389 | -0.948 | 0.877 | 0.280 | 0.092 | 0.081 |
| rs7932354 | -14.583 | 6.017 | 0.015 | -0.449 | 1.752 | 0.798 | 0.075 | 1.580 | 0.962 | -0.148 | 0.536 | 0.783 | 0.030 | 0.794 | 0.970 | 0.074 | 0.103 |
| rs7084921 | 2.221 | 5.676 | 0.696 | 3.023 | 1.655 | 0.068 | -0.077 | 1.511 | 0.959 | -0.267 | 0.511 | 0.601 | 0.112 | 0.758 | 0.882 | 0.192 | 0.116 |
| rs2062377 | -4.081 | 5.678 | 0.472 | -2.094 | 1.637 | 0.201 | 3.684 | 1.504 | 0.014 | 1.229 | 0.508 | 0.015 | -1.908 | 0.755 | 0.012 | 0.181 | 0.119 |
| rs7521902 | -6.316 | 6.562 | 0.336 | -2.045 | 1.906 | 0.283 | 2.000 | 1.761 | 0.256 | 0.595 | 0.596 | 0.318 | -0.813 | 0.885 | 0.358 | 0.203 | 0.128 |
| rs12407028 | -3.456 | 5.680 | 0.543 | 0.472 | 1.633 | 0.773 | -0.709 | 1.503 | 0.637 | -0.299 | 0.506 | 0.555 | 0.300 | 0.753 | 0.691 | 0.215 | 0.132 |
| rs4727338 | -15.145 | 5.945 | 0.011 | 0.162 | 1.726 | 0.925 | 2.651 | 1.586 | 0.095 | 0.539 | 0.535 | 0.314 | -1.042 | 0.795 | 0.190 | 0.038 | 0.157 |
| rs479336 | -17.202 | 6.354 | 0.007 | -2.640 | 1.854 | 0.154 | 3.284 | 1.713 | 0.055 | 0.973 | 0.578 | 0.092 | -1.566 | 0.859 | 0.068 | 0.126 | 0.174 |
| rs7851693 | -10.050 | 5.875 | 0.087 | -3.872 | 1.705 | 0.023 | 1.965 | 1.569 | 0.210 | 0.861 | 0.531 | 0.105 | -1.149 | 0.788 | 0.145 | 0.278 | 0.215 |
| rs227584 | -0.703 | 6.126 | 0.909 | 0.563 | 1.771 | 0.751 | -0.978 | 1.640 | 0.551 | -0.291 | 0.552 | 0.598 | 0.353 | 0.823 | 0.668 | 0.359 | 0.241 |
| rs3790160 | -1.335 | 5.523 | 0.809 | 1.253 | 1.601 | 0.434 | 0.184 | 1.464 | 0.900 | -0.065 | 0.496 | 0.896 | -0.094 | 0.735 | 0.898 | 0.406 | 0.280 |
| rs1864325 | 6.223 | 6.559 | 0.343 | -1.213 | 1.914 | 0.526 | -1.941 | 1.732 | 0.262 | -0.362 | 0.588 | 0.538 | 0.705 | 0.870 | 0.417 | 0.239 | 0.291 |
| rs430727 | 2.567 | 5.682 | 0.651 | -0.320 | 1.649 | 0.846 | -2.247 | 1.506 | 0.136 | -0.628 | 0.509 | 0.217 | 0.998 | 0.755 | 0.186 | 0.323 | 0.293 |
| rs3736228 | -18.961 | 7.631 | 0.013 | -3.144 | 2.232 | 0.159 | 2.076 | 2.016 | 0.303 | 0.602 | 0.685 | 0.380 | -0.876 | 1.015 | 0.388 | 0.183 | 0.303 |
| rs163879 | 13.792 | 5.933 | 0.020 | 1.266 | 1.713 | 0.460 | -0.908 | 1.596 | 0.570 | -0.186 | 0.536 | 0.728 | 0.264 | 0.800 | 0.742 | 0.079 | 0.304 |
| rs4796995 | 6.255 | 5.794 | 0.280 | 3.574 | 1.672 | 0.033 | -1.714 | 1.555 | 0.271 | -0.751 | 0.522 | 0.151 | 0.952 | 0.779 | 0.221 | 0.449 | 0.316 |
| rs4985155 | 14.650 | 5.891 | 0.013 | -0.004 | 1.718 | 0.998 | -0.773 | 1.564 | 0.621 | 0.016 | 0.529 | 0.975 | 0.165 | 0.785 | 0.833 | 0.143 | 0.347 |
| rs1053051 | -2.989 | 5.479 | 0.585 | 2.116 | 1.583 | 0.181 | -1.700 | 1.445 | 0.240 | -0.773 | 0.488 | 0.113 | 0.983 | 0.724 | 0.175 | 0.423 | 0.360 |
| rs13204965 | -13.694 | 6.838 | 0.045 | 0.379 | 1.972 | 0.848 | 0.976 | 1.812 | 0.590 | 0.066 | 0.612 | 0.914 | -0.202 | 0.909 | 0.824 | 0.080 | 0.385 |
| rs12821008 | -7.440 | 5.616 | 0.185 | -1.123 | 1.620 | 0.488 | -1.150 | 1.492 | 0.441 | -0.349 | 0.503 | 0.488 | 0.634 | 0.748 | 0.397 | 0.353 | 0.423 |
| rs3755955 | -14.900 | 7.728 | 0.054 | -3.105 | 2.238 | 0.165 | -0.821 | 2.045 | 0.688 | -0.048 | 0.690 | 0.944 | 0.260 | 1.025 | 0.800 | 0.242 | 0.463 |
| rs6532023 | -2.721 | 5.800 | 0.639 | 2.418 | 1.675 | 0.149 | -1.227 | 1.551 | 0.429 | -0.605 | 0.523 | 0.247 | 0.712 | 0.778 | 0.360 | 0.467 | 0.471 |
| rs6959212 | -11.878 | 5.826 | 0.042 | 1.153 | 1.690 | 0.495 | -1.635 | 1.545 | 0.290 | -0.710 | 0.521 | 0.173 | 1.001 | 0.774 | 0.196 | 0.034 | 0.474 |
| rs4233949 | -6.912 | 5.642 | 0.221 | -2.437 | 1.634 | 0.136 | 1.085 | 1.520 | 0.475 | 0.381 | 0.511 | 0.457 | -0.512 | 0.762 | 0.502 | 0.655 | 0.510 |
| rs1346004 | -10.926 | 5.580 | 0.050 | 2.030 | 1.622 | 0.211 | -0.182 | 1.492 | 0.903 | -0.338 | 0.505 | 0.502 | 0.294 | 0.749 | 0.695 | 0.046 | 0.518 |
| rs7217932 | -0.750 | 5.515 | 0.892 | -2.486 | 1.594 | 0.119 | 1.672 | 1.478 | 0.258 | 0.741 | 0.499 | 0.137 | -0.985 | 0.742 | 0.184 | 0.620 | 0.525 |
| rs7812088 | -2.051 | 8.702 | 0.814 | -3.392 | 2.527 | 0.180 | -0.859 | 2.360 | 0.716 | 0.042 | 0.788 | 0.957 | 0.245 | 1.179 | 0.836 | 0.683 | 0.544 |
| rs10048146 | -11.801 | 7.057 | 0.095 | -2.089 | 2.047 | 0.307 | 0.878 | 1.871 | 0.639 | 0.276 | 0.633 | 0.662 | -0.446 | 0.939 | 0.635 | 0.617 | 0.556 |
| rs7584262 | -5.836 | 6.749 | 0.387 | -1.926 | 1.944 | 0.322 | -0.551 | 1.770 | 0.756 | -0.114 | 0.599 | 0.849 | 0.305 | 0.888 | 0.731 | 0.691 | 0.569 |
| rs7071206 | -13.570 | 6.719 | 0.043 | -1.936 | 1.948 | 0.320 | 0.340 | 1.803 | 0.851 | 0.078 | 0.607 | 0.898 | -0.071 | 0.904 | 0.937 | 0.380 | 0.582 |
| rs13245690 | -8.809 | 5.683 | 0.121 | -1.397 | 1.644 | 0.395 | -1.133 | 1.511 | 0.453 | -0.270 | 0.511 | 0.598 | 0.538 | 0.759 | 0.479 | 0.319 | 0.598 |
| rs7017914 | -5.729 | 5.550 | 0.302 | -0.092 | 1.611 | 0.955 | -1.699 | 1.475 | 0.249 | -0.579 | 0.499 | 0.246 | 0.906 | 0.740 | 0.221 | 0.534 | 0.602 |
| rs1366594 | -6.157 | 5.579 | 0.270 | 0.190 | 1.612 | 0.906 | -1.596 | 1.482 | 0.281 | -0.542 | 0.499 | 0.278 | 0.788 | 0.742 | 0.289 | 0.523 | 0.607 |
| rs3801387 | -14.258 | 6.266 | 0.023 | -1.966 | 1.823 | 0.281 | 2.481 | 1.700 | 0.145 | 0.802 | 0.570 | 0.160 | -1.234 | 0.852 | 0.147 | 0.246 | 0.613 |
| rs10226308 | -17.317 | 7.222 | 0.017 | -1.224 | 2.077 | 0.556 | -0.148 | 1.909 | 0.938 | -0.159 | 0.641 | 0.804 | 0.205 | 0.955 | 0.830 | 0.208 | 0.616 |
| rs4869742 | -6.050 | 6.091 | 0.321 | -2.261 | 1.769 | 0.201 | 0.532 | 1.627 | 0.744 | 0.220 | 0.546 | 0.687 | -0.243 | 0.813 | 0.765 | 0.764 | 0.631 |
| rs7953528 | 4.476 | 7.530 | 0.552 | -2.416 | 2.174 | 0.266 | 1.591 | 2.026 | 0.432 | 0.780 | 0.680 | 0.252 | -1.028 | 1.015 | 0.311 | 0.555 | 0.634 |
| rs17040773 | -1.234 | 7.639 | 0.872 | 0.053 | 2.206 | 0.981 | 0.695 | 1.971 | 0.724 | 0.089 | 0.667 | 0.894 | -0.148 | 0.987 | 0.881 | 0.772 | 0.639 |
| rs1286083 | -11.464 | 7.116 | 0.107 | 1.048 | 2.038 | 0.607 | -0.059 | 1.908 | 0.975 | -0.182 | 0.637 | 0.775 | 0.229 | 0.954 | 0.810 | 0.082 | 0.658 |
| rs3905706 | 0.633 | 6.879 | 0.927 | -1.676 | 1.997 | 0.401 | 1.104 | 1.852 | 0.551 | 0.443 | 0.626 | 0.479 | -0.533 | 0.930 | 0.567 | 0.767 | 0.674 |
| rs6426749 | -13.114 | 7.232 | 0.070 | 0.848 | 2.107 | 0.687 | 1.448 | 1.902 | 0.446 | 0.235 | 0.646 | 0.717 | -0.567 | 0.955 | 0.553 | 0.266 | 0.708 |
| rs9533090 | 0.417 | 5.581 | 0.940 | 2.292 | 1.620 | 0.157 | -1.308 | 1.490 | 0.380 | -0.548 | 0.504 | 0.276 | 0.726 | 0.748 | 0.332 | 0.685 | 0.721 |
| rs344081 | 7.174 | 8.367 | 0.391 | 2.440 | 2.449 | 0.319 | -0.712 | 2.252 | 0.752 | -0.369 | 0.760 | 0.627 | 0.367 | 1.130 | 0.745 | 0.765 | 0.742 |
| rs11755164 | -3.434 | 5.936 | 0.563 | -0.185 | 1.716 | 0.914 | 0.264 | 1.560 | 0.866 | 0.029 | 0.528 | 0.957 | -0.132 | 0.783 | 0.866 | 0.886 | 0.786 |
| rs1564981 | 0.982 | 5.511 | 0.859 | 0.235 | 1.596 | 0.883 | 1.064 | 1.477 | 0.471 | 0.276 | 0.497 | 0.578 | -0.524 | 0.740 | 0.479 | 0.827 | 0.795 |
| rs11623869 | 2.253 | 5.806 | 0.698 | -0.855 | 1.679 | 0.610 | 0.581 | 1.549 | 0.708 | 0.243 | 0.522 | 0.641 | -0.367 | 0.777 | 0.637 | 0.766 | 0.889 |
| rs9466056 | -4.027 | 5.649 | 0.476 | 0.266 | 1.630 | 0.870 | 1.214 | 1.499 | 0.418 | 0.355 | 0.506 | 0.483 | -0.597 | 0.752 | 0.427 | 0.775 | 0.891 |
| rs7108738 | -8.437 | 7.334 | 0.250 | -0.038 | 2.129 | 0.986 | -0.036 | 1.938 | 0.985 | -0.122 | 0.657 | 0.852 | 0.150 | 0.973 | 0.878 | 0.781 | 0.929 |
| rs1373004 | -0.204 | 8.749 | 0.981 | 1.934 | 2.542 | 0.447 | -0.477 | 2.357 | 0.840 | -0.312 | 0.793 | 0.694 | 0.355 | 1.182 | 0.764 | 0.970 | 0.956 |

**Supplementary Table 10:** Association results for the 63 SNPs and BA acquisition. Coefficients (standard error) represent the mean difference in BA (cm^2^) or growth (cm^2^) per year per ‘BMD lowering’ allele.

| SNP | Beta SNP | SE SNP | P SNP | Beta SNP:age | SE SNP:age | P SNP:age | Beta SNP:age^2^ | SE SNP:age^2^ | P SNP:age^2^ | Beta SNP:age^3^ | SE SNP:age^3^ | P SNP:age^3^ | Beta SNP:age^3^ (after 13 years) | SE SNP:age^3^ (after 13 years) | P SNP:age^3^ (after 13 years) | Global Wald | Wald for SNP by age interaction |
| --- | --- | --- | --- | --- | --- | --- | --- | --- | --- | --- | --- | --- | --- | --- | --- | --- | --- |
| rs7521902 | 0.162 | 4.584 | 0.972 | -0.668 | 1.313 | 0.611 | 1.873 | 1.284 | 0.145 | 0.530 | 0.438 | 0.226 | -0.832 | 0.648 | 0.199 | 0.542 | 0.489 |
| rs6426749 | -4.883 | 5.052 | 0.334 | 1.942 | 1.451 | 0.181 | 1.771 | 1.385 | 0.201 | 0.373 | 0.474 | 0.431 | -0.835 | 0.698 | 0.232 | 0.049 | 0.042 |
| rs17482952 | -11.418 | 7.124 | 0.109 | 0.487 | 2.077 | 0.815 | 0.062 | 1.999 | 0.975 | -0.190 | 0.685 | 0.781 | 0.313 | 1.010 | 0.757 | 0.026 | 0.046 |
| rs12407028 | -0.372 | 3.967 | 0.925 | 1.001 | 1.124 | 0.373 | 0.164 | 1.094 | 0.881 | -0.040 | 0.371 | 0.914 | -0.090 | 0.550 | 0.870 | 0.311 | 0.203 |
| rs479336 | -13.016 | 4.438 | 0.003 | -0.863 | 1.276 | 0.499 | 2.755 | 1.247 | 0.027 | 0.729 | 0.424 | 0.086 | -1.271 | 0.628 | 0.043 | 0.030 | 0.067 |
| rs7584262 | -0.686 | 4.715 | 0.884 | -0.066 | 1.338 | 0.961 | -0.822 | 1.290 | 0.524 | -0.239 | 0.440 | 0.587 | 0.415 | 0.649 | 0.523 | 0.961 | 0.924 |
| rs4233949 | -1.384 | 3.941 | 0.725 | -1.331 | 1.125 | 0.237 | 0.454 | 1.106 | 0.681 | 0.203 | 0.375 | 0.589 | -0.213 | 0.557 | 0.702 | 0.722 | 0.584 |
| rs17040773 | 0.245 | 5.333 | 0.963 | -0.014 | 1.517 | 0.992 | 0.126 | 1.435 | 0.930 | -0.091 | 0.489 | 0.852 | 0.117 | 0.721 | 0.871 | 0.616 | 0.510 |
| rs1878526 | 3.965 | 4.743 | 0.403 | 2.288 | 1.361 | 0.093 | -0.192 | 1.310 | 0.883 | -0.082 | 0.447 | 0.854 | 0.030 | 0.660 | 0.964 | 0.199 | 0.123 |
| rs1346004 | -3.873 | 3.898 | 0.320 | 2.413 | 1.116 | 0.031 | -0.692 | 1.086 | 0.524 | -0.427 | 0.370 | 0.249 | 0.486 | 0.547 | 0.375 | 0.131 | 0.275 |
| rs430727 | 3.291 | 3.969 | 0.407 | -0.312 | 1.135 | 0.783 | -0.994 | 1.096 | 0.364 | -0.274 | 0.373 | 0.463 | 0.431 | 0.552 | 0.435 | 0.442 | 0.459 |
| rs1026364 | -4.082 | 4.093 | 0.319 | -2.303 | 1.169 | 0.049 | 1.010 | 1.147 | 0.379 | 0.462 | 0.388 | 0.234 | -0.595 | 0.576 | 0.302 | 0.478 | 0.348 |
| rs344081 | 6.684 | 5.847 | 0.253 | 1.757 | 1.687 | 0.298 | -0.680 | 1.636 | 0.678 | -0.239 | 0.557 | 0.668 | 0.287 | 0.824 | 0.727 | 0.760 | 0.695 |
| rs3755955 | -12.380 | 5.397 | 0.022 | -0.675 | 1.539 | 0.661 | -0.671 | 1.490 | 0.653 | -0.197 | 0.507 | 0.698 | 0.334 | 0.750 | 0.656 | 0.192 | 0.855 |
| rs6532023 | -1.182 | 4.051 | 0.770 | 2.222 | 1.154 | 0.054 | -0.307 | 1.129 | 0.786 | -0.313 | 0.383 | 0.414 | 0.237 | 0.568 | 0.676 | 0.152 | 0.097 |
| rs1366594 | -2.827 | 3.897 | 0.468 | 1.015 | 1.108 | 0.360 | -1.084 | 1.079 | 0.315 | -0.430 | 0.366 | 0.241 | 0.574 | 0.542 | 0.290 | 0.629 | 0.678 |
| rs9466056 | -1.326 | 3.946 | 0.737 | 1.079 | 1.122 | 0.336 | 1.065 | 1.092 | 0.329 | 0.250 | 0.371 | 0.500 | -0.504 | 0.549 | 0.359 | 0.450 | 0.352 |
| rs11755164 | -1.652 | 4.146 | 0.690 | -0.248 | 1.180 | 0.833 | 1.144 | 1.135 | 0.314 | 0.336 | 0.387 | 0.386 | -0.580 | 0.572 | 0.310 | 0.773 | 0.642 |
| rs13204965 | -3.769 | 4.777 | 0.430 | 1.544 | 1.357 | 0.255 | 0.056 | 1.319 | 0.966 | -0.144 | 0.449 | 0.748 | 0.113 | 0.664 | 0.865 | 0.536 | 0.614 |
| rs4869742 | -3.805 | 4.255 | 0.371 | -0.236 | 1.218 | 0.846 | 1.145 | 1.185 | 0.334 | 0.277 | 0.401 | 0.489 | -0.473 | 0.594 | 0.427 | 0.853 | 0.774 |
| rs7751941 | 1.770 | 4.611 | 0.701 | -2.157 | 1.318 | 0.102 | -1.539 | 1.276 | 0.228 | -0.254 | 0.434 | 0.559 | 0.686 | 0.642 | 0.286 | 0.039 | 0.020 |
| rs10226308 | -13.470 | 5.043 | 0.008 | -0.685 | 1.428 | 0.632 | 0.325 | 1.387 | 0.815 | 0.025 | 0.470 | 0.958 | -0.059 | 0.697 | 0.932 | 0.159 | 0.740 |
| rs6959212 | -6.066 | 4.068 | 0.136 | 2.020 | 1.163 | 0.082 | -3.036 | 1.122 | 0.007 | -1.059 | 0.382 | 0.006 | 1.587 | 0.564 | 0.005 | 0.005 | 0.081 |
| rs4727338 | -8.101 | 4.153 | 0.051 | 1.383 | 1.189 | 0.245 | 0.559 | 1.155 | 0.628 | -0.092 | 0.392 | 0.815 | -0.016 | 0.581 | 0.977 | 0.047 | 0.108 |
| rs13245690 | -4.153 | 3.970 | 0.296 | 0.354 | 1.132 | 0.754 | -1.826 | 1.101 | 0.097 | -0.553 | 0.375 | 0.141 | 0.891 | 0.555 | 0.108 | 0.267 | 0.534 |
| rs3801387 | -6.784 | 4.377 | 0.121 | -0.251 | 1.255 | 0.841 | 1.721 | 1.239 | 0.165 | 0.504 | 0.419 | 0.229 | -0.837 | 0.623 | 0.179 | 0.525 | 0.665 |
| rs7812088 | 5.087 | 6.075 | 0.402 | -1.759 | 1.740 | 0.312 | -2.246 | 1.718 | 0.191 | -0.379 | 0.578 | 0.513 | 0.880 | 0.861 | 0.307 | 0.302 | 0.210 |
| rs7017914 | -2.813 | 3.877 | 0.468 | 0.802 | 1.110 | 0.470 | -1.228 | 1.074 | 0.253 | -0.452 | 0.366 | 0.217 | 0.653 | 0.541 | 0.227 | 0.705 | 0.808 |
| rs2062377 | -1.998 | 3.965 | 0.614 | -1.308 | 1.126 | 0.245 | 2.557 | 1.096 | 0.020 | 0.823 | 0.373 | 0.027 | -1.312 | 0.552 | 0.017 | 0.137 | 0.092 |
| rs7851693 | -3.078 | 4.103 | 0.453 | -1.643 | 1.175 | 0.162 | 0.984 | 1.142 | 0.389 | 0.479 | 0.390 | 0.219 | -0.648 | 0.576 | 0.261 | 0.492 | 0.386 |
| rs3905706 | 2.637 | 4.805 | 0.583 | -0.850 | 1.375 | 0.536 | 0.021 | 1.346 | 0.988 | 0.038 | 0.458 | 0.934 | 0.020 | 0.678 | 0.977 | 0.788 | 0.816 |
| rs1373004 | 0.087 | 6.109 | 0.989 | 0.855 | 1.749 | 0.625 | 0.620 | 1.718 | 0.718 | 0.105 | 0.583 | 0.857 | -0.256 | 0.865 | 0.767 | 0.982 | 0.949 |
| rs7071206 | -8.692 | 4.692 | 0.064 | -0.984 | 1.343 | 0.464 | -0.054 | 1.313 | 0.967 | -0.009 | 0.446 | 0.984 | 0.065 | 0.661 | 0.922 | 0.462 | 0.789 |
| rs7084921 | 0.321 | 3.964 | 0.936 | 1.695 | 1.140 | 0.137 | 1.676 | 1.101 | 0.128 | 0.336 | 0.375 | 0.370 | -0.749 | 0.554 | 0.177 | 0.057 | 0.031 |
| rs7108738 | -2.991 | 5.123 | 0.559 | 1.992 | 1.465 | 0.174 | 0.629 | 1.410 | 0.656 | -0.075 | 0.482 | 0.876 | -0.111 | 0.710 | 0.876 | 0.508 | 0.406 |
| rs10835187 | -12.498 | 3.862 | 0.001 | -1.186 | 1.105 | 0.283 | 0.442 | 1.070 | 0.680 | 0.114 | 0.366 | 0.755 | -0.162 | 0.540 | 0.764 | 0.044 | 0.471 |
| rs163879 | 8.536 | 4.145 | 0.039 | 0.257 | 1.180 | 0.828 | -0.340 | 1.162 | 0.770 | -0.039 | 0.394 | 0.921 | 0.067 | 0.584 | 0.908 | 0.317 | 0.752 |
| rs7932354 | -8.786 | 4.203 | 0.037 | 0.561 | 1.206 | 0.642 | -0.005 | 1.151 | 0.996 | -0.143 | 0.394 | 0.716 | 0.043 | 0.580 | 0.941 | 0.045 | 0.091 |
| rs3736228 | -8.682 | 5.328 | 0.103 | -0.712 | 1.538 | 0.644 | -1.377 | 1.470 | 0.349 | -0.421 | 0.503 | 0.403 | 0.751 | 0.742 | 0.311 | 0.175 | 0.364 |
| rs2887571 | -7.265 | 4.524 | 0.108 | -0.608 | 1.315 | 0.644 | 2.545 | 1.243 | 0.041 | 0.807 | 0.426 | 0.058 | -1.352 | 0.627 | 0.031 | 0.028 | 0.024 |
| rs7953528 | 6.804 | 5.257 | 0.196 | -2.448 | 1.496 | 0.102 | 1.569 | 1.474 | 0.287 | 0.818 | 0.499 | 0.101 | -1.067 | 0.741 | 0.150 | 0.111 | 0.181 |
| rs12821008 | -5.360 | 3.922 | 0.172 | -0.492 | 1.115 | 0.659 | -1.068 | 1.087 | 0.326 | -0.349 | 0.369 | 0.344 | 0.608 | 0.547 | 0.266 | 0.176 | 0.231 |
| rs2016266 | -12.573 | 4.082 | 0.002 | -0.932 | 1.164 | 0.424 | 1.001 | 1.129 | 0.376 | 0.291 | 0.385 | 0.449 | -0.439 | 0.569 | 0.440 | 0.072 | 0.756 |
| rs736825 | 5.073 | 4.295 | 0.238 | -1.937 | 1.231 | 0.116 | -0.931 | 1.175 | 0.428 | -0.020 | 0.402 | 0.960 | 0.200 | 0.592 | 0.736 | 0.031 | 0.035 |
| rs1053051 | -0.380 | 3.826 | 0.921 | 3.024 | 1.088 | 0.005 | -1.619 | 1.051 | 0.124 | -0.799 | 0.358 | 0.026 | 0.962 | 0.529 | 0.069 | 0.051 | 0.031 |
| rs9533090 | 0.454 | 3.898 | 0.907 | 1.631 | 1.115 | 0.144 | -0.527 | 1.085 | 0.627 | -0.244 | 0.369 | 0.510 | 0.292 | 0.546 | 0.593 | 0.682 | 0.613 |
| rs1286083 | -3.151 | 4.971 | 0.526 | 1.635 | 1.401 | 0.243 | -1.464 | 1.389 | 0.292 | -0.485 | 0.467 | 0.300 | 0.763 | 0.697 | 0.274 | 0.173 | 0.373 |
| rs11623869 | 1.405 | 4.054 | 0.729 | -1.404 | 1.155 | 0.224 | 0.716 | 1.128 | 0.526 | 0.317 | 0.383 | 0.407 | -0.389 | 0.567 | 0.493 | 0.771 | 0.781 |
| rs9921222 | -7.289 | 3.805 | 0.055 | -0.737 | 1.090 | 0.499 | 0.204 | 1.068 | 0.848 | 0.044 | 0.363 | 0.904 | -0.074 | 0.538 | 0.890 | 0.503 | 0.741 |
| rs13336428 | 2.822 | 3.983 | 0.479 | 2.059 | 1.140 | 0.071 | -1.080 | 1.106 | 0.329 | -0.424 | 0.377 | 0.261 | 0.504 | 0.557 | 0.366 | 0.198 | 0.120 |
| rs4985155 | 9.979 | 4.114 | 0.015 | -1.508 | 1.183 | 0.203 | 0.014 | 1.140 | 0.991 | 0.296 | 0.388 | 0.446 | -0.243 | 0.574 | 0.672 | 0.047 | 0.200 |
| rs1564981 | 0.802 | 3.849 | 0.835 | -0.007 | 1.099 | 0.995 | 1.496 | 1.075 | 0.164 | 0.437 | 0.365 | 0.231 | -0.738 | 0.541 | 0.172 | 0.638 | 0.562 |
| rs1566045 | 0.133 | 4.837 | 0.978 | 1.425 | 1.388 | 0.305 | -2.795 | 1.368 | 0.041 | -0.905 | 0.464 | 0.051 | 1.308 | 0.689 | 0.058 | 0.094 | 0.061 |
| rs10048146 | -7.876 | 4.931 | 0.110 | -1.167 | 1.411 | 0.408 | 0.437 | 1.364 | 0.749 | 0.127 | 0.465 | 0.784 | -0.178 | 0.687 | 0.796 | 0.682 | 0.772 |
| rs4790881 | -6.047 | 4.179 | 0.148 | -1.102 | 1.191 | 0.355 | -2.166 | 1.154 | 0.061 | -0.627 | 0.392 | 0.110 | 1.100 | 0.580 | 0.058 | 0.020 | 0.027 |
| rs4792909 | -4.801 | 4.069 | 0.238 | -1.118 | 1.168 | 0.339 | 1.441 | 1.126 | 0.200 | 0.500 | 0.384 | 0.192 | -0.813 | 0.567 | 0.152 | 0.133 | 0.084 |
| rs227584 | -0.845 | 4.279 | 0.844 | -0.427 | 1.219 | 0.726 | -0.666 | 1.194 | 0.577 | -0.098 | 0.405 | 0.809 | 0.203 | 0.601 | 0.736 | 0.737 | 0.639 |
| rs1864325 | 4.714 | 4.579 | 0.303 | -2.357 | 1.319 | 0.074 | 0.334 | 1.262 | 0.791 | 0.360 | 0.431 | 0.404 | -0.390 | 0.636 | 0.540 | 0.127 | 0.194 |
| rs7217932 | 1.531 | 3.853 | 0.691 | -1.347 | 1.097 | 0.220 | 0.859 | 1.076 | 0.425 | 0.459 | 0.366 | 0.210 | -0.565 | 0.542 | 0.297 | 0.618 | 0.494 |
| rs4796995 | 4.706 | 4.047 | 0.245 | 2.407 | 1.152 | 0.037 | -0.886 | 1.131 | 0.434 | -0.428 | 0.383 | 0.264 | 0.502 | 0.568 | 0.377 | 0.408 | 0.310 |
| rs884205 | -2.175 | 4.510 | 0.630 | 0.413 | 1.292 | 0.749 | 2.572 | 1.273 | 0.043 | 0.621 | 0.432 | 0.150 | -1.044 | 0.641 | 0.103 | 0.027 | 0.013 |
| rs10416218 | 8.530 | 4.501 | 0.058 | -1.389 | 1.298 | 0.284 | 0.124 | 1.272 | 0.922 | 0.298 | 0.433 | 0.491 | -0.348 | 0.641 | 0.587 | 0.021 | 0.061 |
| rs3790160 | -0.720 | 3.858 | 0.852 | 0.927 | 1.102 | 0.400 | -0.386 | 1.066 | 0.717 | -0.222 | 0.364 | 0.541 | 0.235 | 0.537 | 0.662 | 0.862 | 0.762 |
